# c-JUN enhances CRISPR knockin anti-B7-H3 CAR T cell function in small cell lung cancer and thoracic SMARCA4-deficient undifferentiated tumors

**DOI:** 10.1016/j.xcrm.2025.102549

**Published:** 2026-01-20

**Authors:** Hyatt Balke-Want, Vimal Keerthi, Maria Del Carmen Arenas, Yiyun Chen, Meena Malipatlolla, Dorota D. Klysz, Peng Xu, Katie Ho, Kyle Asano, David Stahl, Jing Huang, Aidan Retherford, Sunny Patel, Carley Fowler, Lukas Maas, Nikolaos Gkitsas-Long, Qiaoshi Jiang, Xikun Liu, Roland Ullrich, Julie George, Sabine Heitzeneder, Ramya Tunuguntla, Julien Sage, Elena Sotillo, Crystal L. Mackall, Steven A. Feldman

**Affiliations:** 1 Center for Cancer Cell Therapy, Stanford Cancer Institute, Stanford University, Stanford, CA, USA; 2 Department I of Internal Medicine, Center for Integrated Oncology Aachen Bonn Cologne Duesseldorf, University of Cologne, Medical Faculty and University Hospital Cologne, Cologne, Germany; 3 Cancer Center Cologne Essen (CCCE), Cologne, Germany; 4 Center for Molecular Medicine Cologne (CMMC), University of Cologne, Cologne, Germany; 5 University of Cologne, Faculty of Medicine and University Hospital Cologne, Department of Translational Genomics, Cologne, Germany; 6 Departments of Pediatrics and Genetics, Stanford University School of Medicine, Stanford, CA 94305, USA; 7 Weill West Coast Cancer Hub, Stanford, CA, USA; 8 Parker Institute for Cancer Immunotherapy, San Francisco, CA, USA; 9 Ludwig Center for Cancer Stem Cell Research and Medicine, Stanford University School of Medicine, Stanford, CA, USA; 10 Division of Pediatric Hematology/Oncology and Division of Stem Cell Transplantation and Regenerative Medicine, Department of Pediatrics, Stanford University School of Medicine, Stanford, CA, USA; 11 Division of Blood and Marrow Transplantation and Cell Therapy, Department of Medicine, Stanford University School of Medicine, Stanford, CA, USA; 12 Laboratory for Cell and Gene Medicine, Stanford University School of Medicine, Stanford, CA, USA

**Keywords:** small cell lung cancer, thoracic SMARCA4-deficient undifferentiated tumor, B7-H3, transforming growth factor-beta 1, non-viral CAR T-cells, CRISPR knock-in, cell therapy scaling

## Abstract

Small cell lung cancer (SCLC), a highly lethal disease, limits T cell responses by downregulating major histocompatibility (MHC) class I molecules. Because chimeric antigen receptor (CAR) T cells are not MHC restricted, they may provide a powerful strategy against SCLC. However, few CAR targets for SCLC are known. Here, we show that B7-H3/*CD276* is expressed in SCLC and thoracic SMARCA4-deficient undifferentiated tumors (UTs) that can clinicopathologically mimic SCLC. Thoracic SMARCA4-deficient UTs limit killing by B7-H3 CAR T cells via secretion of transforming growth factor β1 (TGF-β1). To overcome tumor-driven CAR T cell suppression, we knock in c-JUN alongside a B7-H3 CAR into the *TRAC* locus of primary human T cells utilizing CRISPR-Cas9. Non-viral c-JUN+B7-H3 CAR T cells show enhanced killing of both SCLC cells with low antigen density and thoracic SMARCA4-deficient UTs, providing a platform to address these highly aggressive entities. We also provide evidence that good manufacturing practice (GMP) clinical-scale manufacturing is feasible for c-JUN+B7-H3 CAR T cells.

## Highlights

• B7-H3 can be targeted in SCLC and thoracic SMARCA4-deficient UTs with CAR T cells
• TGF-β1 drives resistance in thoracic SMARCA4-deficient UTs
• c-JUN enhances killing of B7-H3 CAR T cells by inducing type I/II cytokines
• CRISPR knockin of c-JUN+B7-H3 CAR (3.6 kb) is feasible at the clinical scale

## Graphical abstract

**Figure:**
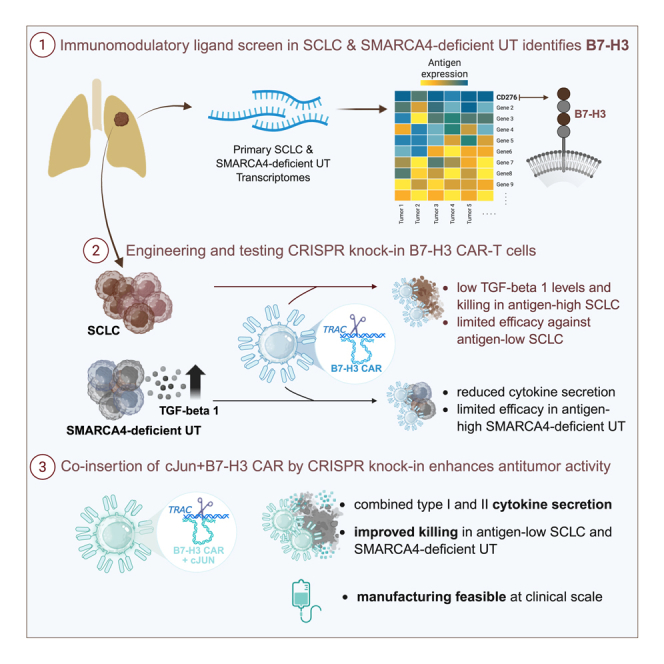

## Teaser

Balke-Want et al. show that B7-H3 is an actionable CAR target in SCLC and thoracic SMARCA4-deficient UTs. However, in thoracic SMARCA4-deficient UTs, TGF-β1 suppresses their activity. By engineering CRISPR knockin c-JUN+B7-H3 CAR T cells with combined expression of type I/II cytokines, the authors overcome TGF-β1-mediated suppression.

## Introduction

Small cell lung cancer (SCLC) accounts for 10%–15% of all lung cancer cases, and despite an initial response to platinum-based chemotherapy, >90% of patients with extensive disease will relapse within 2 years after treatment. Recently, immune checkpoint inhibitors (ICIs) targeting PD1 have been approved for the treatment of SCLC.^1^ Despite a high tumor mutational burden caused by combined bi-allelic loss of *TP53* and *Rb1*,^2^^,^^3^ the efficacy of checkpoint inhibitors remains poor.^4^^,^^5^ SCLC has been shown to express low levels of *TAP* and *B2M*, which are needed for antigen loading and presentation via the major histocompatibility (MHC) class I complex.^6^ Thus, enabling tumor cells to evade a T cell receptor (TCR)-mediated T cell attack, which provides a rational for the limited clinical activity of checkpoint inhibition in SCLC. In contrast, chimeric antigen receptors (CARs) enable T cells to kill tumor cells in a TCR-independent manner.^7^ However, in SCLC, the number of CAR targets reported so far is limited to CD56 and DLL3,^8^^,^^9^ and both are mainly expressed in neuroendocrine (NE) SCLC.^8^^,^^10^

Intratumoral heterogeneity is a major driver of resistance in SCLC,^11^^,^^12^ with non-NE cells enhancing resistance against chemotherapy by promoting growth of the NE population.^13^ Therefore, novel targets expressed across SCLC subpopulations are urgently needed for the development of CAR T cells in SCLC. Recent work indicates that B7-H3, a member of the B7 ligand family that can be detected in many cancers,^14^ is expressed broadly in SCLC.^15^ We and others have found CAR T cells against B7-H3 to be effective in multiple tumor types^16^^,^^17^^,^^18^^,^^19^ and therefore developed clinical trials to credential B7-H3 CAR T cells.^20^ Hence, expanding the scope of B7-H3 CAR T cells to target SCLC provides an exciting and swiftly translatable therapeutic opportunity for this otherwise deadly disease.

Classification of SCLC has been based on the expression of the lineage-defining transcription factors *ASCL1*, *NEUROD1*, *POU2F3*, and *YAP1*. While *ASCL1+* and *NEUROD1+* are considered key NE transcription factors, *POU2F3* and *YAP1* expression has defined the non-NE SCLC subtype.^10^ However, lately, the existence of YAP1+ SCLC has been challenged, since later studies failed to detect YAP1 in an exclusive manner.^12^^,^^21^ Also, very recent work has identified that many YAP1+ cell lines commonly used by the SCLC community are no longer considered as SCLC but rather are thoracic SMARCA4-deficient undifferentiated tumors (UTs),^22^ a newly classified entity with sparse clinical data available.^23^ Furthermore, they present as aggressive tumors, with most patients harboring already metastasized disease (in ∼80%), and poorly respond to conventional chemo-immunotherapy.^24^ Thus, utilizing available cell line models of thoracic SMARCA4-deficient UTs, previously falsely classified as YAP+ SCLC,^22^ provides an opportunity to develop novel immunotherapies against this fatal disease.

The production of good manufacturing practice (GMP) viral vectors needed for CAR transgene delivery during human CAR T cell manufacturing incurs a high cost and is associated with extensive biosafety testing, creating a bottleneck for the application of CAR T cells to patients.^25^ Thus, we and others have previously developed approaches to manufacture non-viral CRISPR-Cas9 knockin (CKI) CAR T cells.^26^^,^^27^^,^^28^^,^^29^ While different types of DNA templates can be utilized (double-stranded DNA [dsDNA], single-stranded DNA [ssDNA], or nanoplasmid [NP] DNA), we opted for NP DNA, which can be generated at yields sufficient to meet the demands of both early clinical trials^30^ and manufacturing post-approval.^31^

In this study, we compare RNA expression levels of various immunomodulatory ligands and identify *CD276* (encodes for B7-H3) overexpression in primary human SCLC and thoracic SMARCA4-deficient UTs. Interestingly, *CD276*, unlike other clinically relevant targets (*DLL3* and *SEZ6*), is expressed across the NE and non-NE SCLC subtypes. In cell line models, we confirm the expression of B7-H3, albeit with some heterogeneity across the tested cell lines, at levels amenable to CAR T cell-mediated killing. Given the large, combined number of patients with SCLC and thoracic SMARCA4-deficient UTs, we developed a non-viral approach to generate B7-H3 CAR T cells utilizing targeted insertion, which was robust enough for co-insertion of the AP-1 transcription factor c-JUN to further enhance potency in SCLC with low antigen density and to potentially prevent exhaustion in the largely immunosuppressive SCLC tumor microenvironment. However, despite comparably high levels of B7-H3, the thoracic SMARCA4-deficient UT is resistant against B7-H3 CAR T cells, which is governed by transforming growth factor β1 (TGF-β1). To overcome TGF-β1-mediated suppression in SMARCA4-deficient UTs, we tested B7-H3 CAR T cells co-expressing c-JUN and found that c-JUN boosts potency in thoracic SMARCA4-deficient UTs by inducing a combined type 1/2 cytokine profile and preventing B7-H3 CAR T cell exhaustion, as indicated by reduced levels of NR4A2 expression. Importantly, we provide evidence that our non-viral manufacturing process, which utilizes GMP-compatible reagents, is feasible at the clinical scale and that final products are free from off-target insertion.

## Results

### B7-H3 is a relevant CAR target in SCLC and thoracic SMARCA4-deficient UT

**Figure 1:**
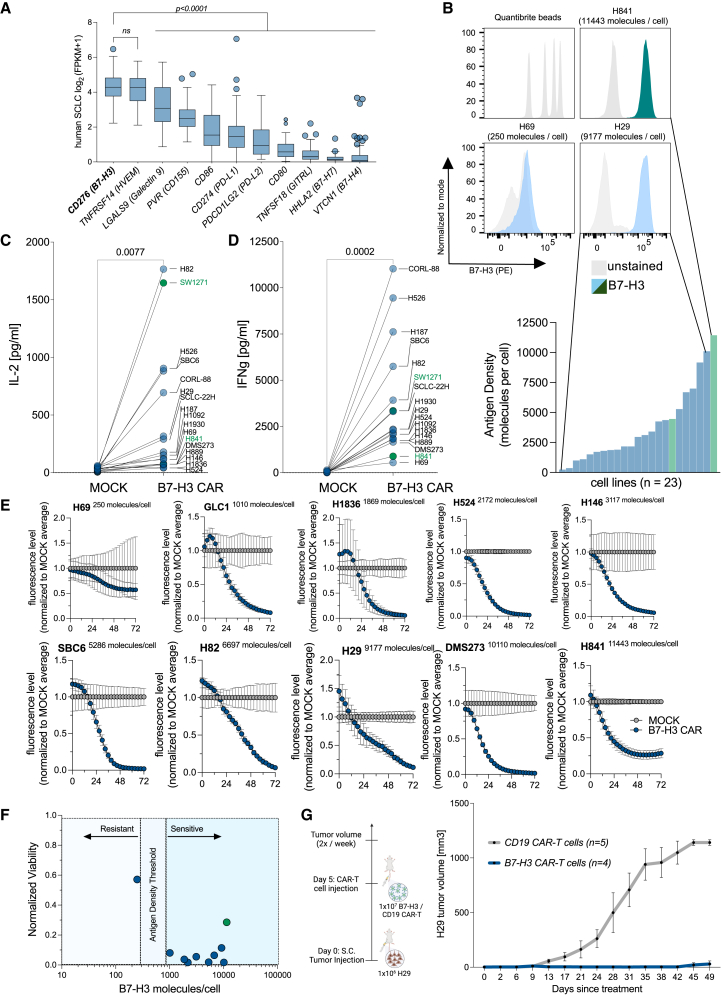
Identification of B7-H3 and screening of viral transduced anti-B7-H3 CAR T cells in SCLC and thoracic SMARCA4-deficient UTs (A) RNA expression levels of relevant immunomodulatory ligands in primary human SCLC (from George et al.^2^, *n* = 81). Statistical differences were determined using two-way ANOVA and Dunnett’s multiple comparison test. (B) Flow cytometric analysis of B7-H3 expression and B7-H3 quantification in human SCLC and thoracic SMARCA4-deficient UT cell lines. Shown are representative histograms (top) and antigen density levels (bottom) for all screened cell lines. B7-H3 surface molecules on tumor cell lines were estimated by Quantibrite beads, which are provided with a known molecule density per peak to allow for antigen quantification. (C and D) Pooled IL-2 (C) and IFNγ (D) secretion detected via ELISA after 24 h of co-culture (E:T = 1:1) of viral transduced anti-B7-H3 CAR T cells with SCLC (blue) and thoracic SMARCA4-deficient UT (green) cell lines (*n* = 17). Statistical differences were assessed using two-tailed and paired *t* tests. (E) Normalized tumor GFP fluorescence levels over time (in hours) after co-culture of respective cell lines with either MOCK or anti-B7-H3 CAR T cells (E:T = 1:1). Tumor cell viability was monitored using the Incucyte live-cell imaging system. Error bars indicate the SD. (F) Dot plot of normalized viability after 72 h of co-culture (E:T = 1:1) with GFP-positive SCLC (blue) and thoracic SMARCA4-deficient UT (green) cell lines (*n* = 10) and B7-H3 antigen levels from (B). (G) *In vivo* treatment of NJH29 SCLC with either anti-B7-H3 or anti-CD19 CAR T cells. Error bars indicate the SD.

To identify actionable immunotherapeutic targets in SCLC, we analyzed the RNA expression levels of 11 highly relevant T cell modulatory ligands^32^^,^^33^ in a previously published dataset of 81 primary human SCLC samples.^2^ When we ranked them by level of expression, we identified *CD276* (which encodes for B7-H3) and *TNFRSF14* (HVEM) as the top overexpressed immunomodulatory ligands (Figure 1A). We also confirmed that B7-H3 is highly expressed compared to other T cell modulatory ligands at the protein level in five human SCLC cell lines, which were recently screened against a total of 332 surface markers^34^ (Figure S1A). Next, we quantified the abundance of B7-H3 using a Quantibrite-based flow cytometry assay^35^^,^^36^ across 21 human SCLC cell lines representative of the genomic heterogeneity of SCLC. Established genomic lesions within our cell line panel included, but were not limited to, amplifications in *MYC*, *MYCN*, and *MYCL*, deletions of *PTEN*, and mutations in *NRAS*.^13^^,^^37^^,^^38^^,^^39^ We also included two thoracic SMARCA4-deficient UT cell lines (H841 and SW1271), which have recently been reclassified from YAP1+ non-NE SCLC.^22^ We identified that 22/23 cell lines express >1,000 molecules/cell on their surface and a median molecule density of 2,966 molecules/cell across the tested panel. Interestingly, thoracic SMARCA4-deficient UT cell lines showed high levels of B7-H3 expression (4,463 and 11,443 molecules/cell, respectively) (Figures 1B and S1B), a finding corroborated in the transcriptomic data from primary human thoracic SMARCA4-deficient UTs^40^ (Figure S1C). Intratumoral heterogeneity is a driver of resistance in SCLC against chemotherapy^11^; thus, we tested whether *Cd276* expression changes over time in the Myc-driven RPM (Rb1^fl/fl^Trp53^fl/fl^Myc^LSL/LSL)^ mouse model, which has previously been used as a model to recapitulate subtype plasticity in SCLC.^41^ Importantly, while the expression of NE markers, as well as currently investigated targets in SCLC, namely *Dll3* and *Sez6*, decreased and the expression of non-NE markers increased, *Cd276* showed constant expression over time (Figure S1D). We validated the finding that *CD276* is expressed across the NE and non-NE SCLC subtypes in transcriptomic data from primary human SCLC and further showed that *CD276* was more highly expressed than *DLL3* and *SEZ6* in non-NE SCLC (Figures S1E and S1F).

Given the high expression of B7-H3 in SCLC and thoracic SMARCA4-deficient UTs, we wondered if the levels of expression were sufficient to activate B7-H3 CAR T cells. To this end, we co-cultured B7-H3 CAR T cells against 15 SCLC as well as 2 thoracic SMARCA4-deficient UT cell lines and assessed IL-2 and IFNγ secretion via ELISA. We observed statistically significant IL-2 and IFNγ production across all tested cell lines after 24 h of co-culture (Figures 1C and 1D). We next selected 10 cell lines expressing the full range of B7-H3 protein densities (250–11,443 molecules/cell) and evaluated killing by the B7-H3 CAR using live-cell imaging. Tumor viability data indicated that antigen density predicted the killing of the B7-H3 CAR, which is in line with our previous work,^16^^,^^35^^,^^42^^,^^43^^,^^44^ and we showed that the antigen density threshold required for killing at a 1:1 effector-to-target (E:T) ratio is approximately between 250 and 1,000 molecules/cell of B7-H3 (Figures 1E and 1F). Finally, we found that B7-H3 CAR T cells prevent tumor growth more efficiently as compared to control anti-CD19 CAR T cells when tested *in vivo* utilizing the NJH29 SCLC cell line model (Figure 1G).^45^

### CRISPR-mediated knockin of a B7-H3 CAR is feasible, but efficacy is limited to SCLC

**Figure 2:**
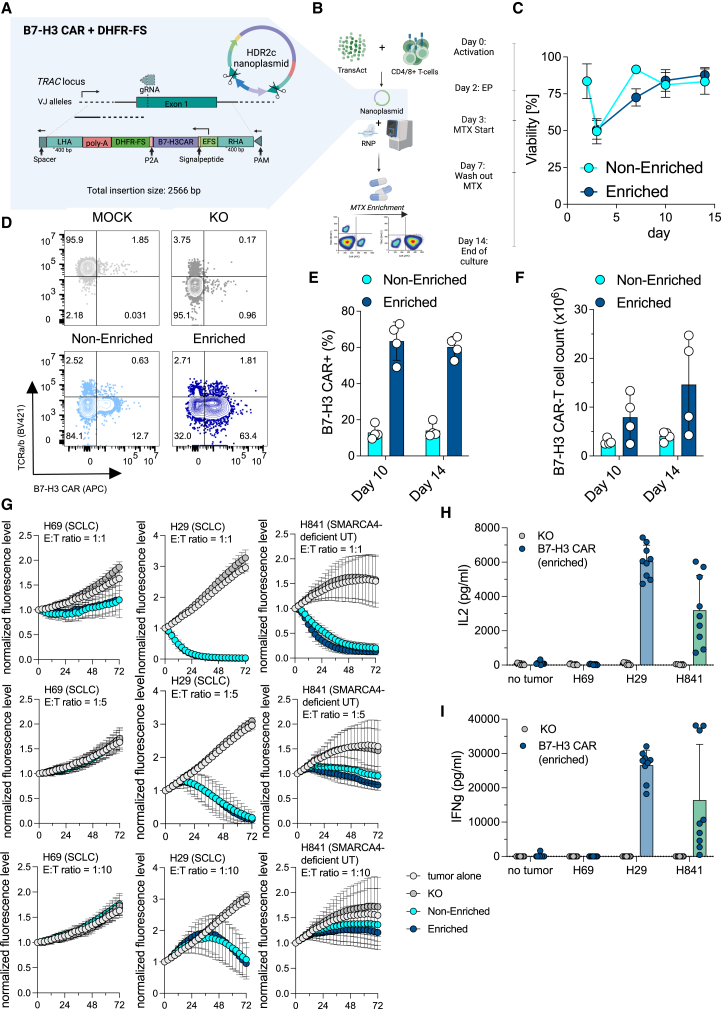
Generation and functional testing of non-viral CKI B7-H3 CAR T cells in SCLC and thoracic SMARCA4-deficient UTs (A and B) Schematic representation of NP DNA constructs (A) utilized for HDR-mediated CKI of a B7-H3 CAR+DHFR-FS construct and workflow for CEMENT using MTX (B). (C) Viabilities for non-enriched and enriched B7-H3 CAR T cells over time. (D) Representative quadrant plots indicating fractions of CRISPR knockout and knockin populations. (E and F) CKI frequencies (E) and yields (F) for B7-H3 CAR+ T cells as determined on days 10 and 14 post-activation are shown. All experiments were conducted with *n* = 4 independent donors utilizing 1 × 10^7^ T cells for electroporation per sample. (G) Tumor cell killing in H69, NJH29, and H841 cell lines using indicated E:T ratios (*n* = 4 independent donors, using technical triplicates per donor). (H and I) IL-2 (H) and IFNγ (I) ELISA from supernatant harvested 24 h post-co-culture of B7-H3 CAR T cells (*n* = 3 independent donors) and indicated cell lines at an E:T ratio of 1:1. Error bars indicate the SD.

To overcome cumbersome and costly production of a GMP-grade viral vector for the potential translation of our B7-H3 CAR in SCLC, we established a non-viral protocol for targeted insertion of the B7-H3 CAR into the bona fide *TRAC* locus via CKI. We utilized NP DNA, which can be manufactured at yields sufficient to support large clinical trials and manufacturing post-approval, to deliver the desired payload.^25^^,^^31^ When comparing the insertion of the B7-H3 CAR via homology-directed repair (HDR) versus homology-independent targeted insertion (HITI), we identified HDR to result in comparable insertion efficiencies (∼10%–15%) but, on average, 4-fold higher B7-H3 CAR+ cell yields (Figures S2A–S2D). To facilitate downstream enrichment of successfully edited B7-H3 CAR T with methotrexate (MTX),^28^ we designed an NP DNA template for the CKI of B7-H3 CAR, along with a mutant version of the human dihydrofolate reductase (DHFR-FS). In addition to the transgenes, our templates consisted of homology arms (each ∼400 bp in length), an EF1a promoter-derived short version (EFS), and a poly(A) signal. B7-H3 CAR and DHFR-FS transgenes were separated by a P2A cleavage site, and the NP DNA template incorporated 2 internal RNP cut sites for linearization (Figure 2A). Next, we designed a process utilizing CRISPR knockin enrichment (CEMENT) post-editing, relying on the DHFR-FS/MTX system, which we recently optimized for seamless integration into a 14-day process^28^ (Figure 2B). We tested our approach across 4 independent donors and found a drop in total T cell viability from 83.5% on day 2 to 50% on day 3, which recovered by day 7 in non-enriched and by day 10 in enriched cultures, respectively (Figure 2C). B7-H3 CAR CKI efficiencies were 13%–14%, and CEMENT resulted in an average 4.5-fold enrichment of B7-H3 CAR+ T cells (Figures 2D and 2E). Given the toxicity associated with electroporation of DNA,^26^^,^^30^ we assessed the growth of B7-H3 CAR T cells from days 10 to 14 and observed increasing cell yields, indicating the recovery of CKI B7-H3 CAR T cells (Figure 2F). Finally, we tested CKI B7-H3 CAR T cells *in vitro* against the B7-H3 ultralow H69 (250 molecules/cell) and two B7-H3-positive cell lines: NJH29 (SCLC, 9,177 molecules/cell) and H841 (thoracic SMARCA4-deficient UT, 11,443 molecules/cell). Surprisingly, we found that B7-H3 CAR T cells kill H841 cells less efficiently *in vitro* when E:T ratios were lowered (Figure 2G) and secrete on average ∼50% and 40% less IL-2 and IFNγ, respectively, as compared to NJH29 (Figures 2H and 2I). Furthermore, *in vivo* assessment of NJH29 and H841 xenograft mice recapitulated discrepancies in response to CKI B7-H3 CAR T cells (Figures S2E–S2G).

### Concomitant knockin of c-JUN is feasible and results in enhanced activity of B7-H3 CAR T cells against low-antigen SCLC

**Figure 3:**
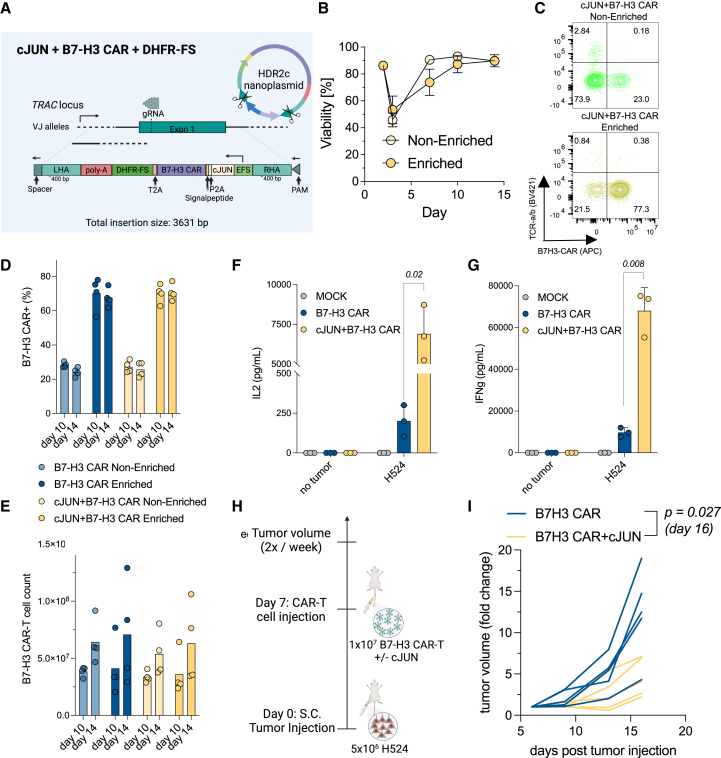
Efficient generation of c-JUN+B7-H3 CAR T cells via CKI with improved function in antigen-low SCLC (A) Schematic representation of NP DNA constructs utilized for HDR-mediated CKI of c-JUN+B7-H3 CAR+DHFR-FS construct. (B) Viabilities for non-enriched and enriched c-JUN+B7-H3 CAR T cells over time. (C) Representative quadrant plots indicating fractions of CRISPR knockout and knockin populations. (D and E) CKI frequencies (D) and yields (E) for B7-H3 CAR+ T cells with or without c-JUN as determined on days 10 and 14 post-activation. All experiments were conducted with *n* = 4 independent donors utilizing 2 × 10^7^ T cells for electroporation per sample. (F and G) IL-2 (F) and IFNγ (G) secretion after 24 h of co-culture at an E:T ratio of 1:1. Statistical differences were assessed using two-tailed and paired *t* tests. One representative out of five donors is shown here. Pooled data for all five donors are provided in Figures S3A and S3B. (H) Experimental setup for *in vivo* treatment of H524 low-antigen SCLC tumors. (I) Tumor volumes indicated as fold change over time post-tumor injection. A two-tailed, unpaired *t* test was used. Error bars indicate the SD.

We have recently identified that the AP-1 factor c-JUN rescues CAR T cells from exhaustion^46^ and enhances killing in antigen-low tumors.^35^ Given the broad but variable expression levels of B7-H3 in SCLC, we next wondered if co-insertion of c-JUN via CKI enhances B7-H3 CAR T cell activity in antigen-low SCLC. Thus, we designed another NP DNA template for targeted insertion into *TRAC*, which incorporated the c-JUN sequence, followed by sequences for P2A-B7-H3 CAR-T2A-DHFR-FS. This construct had a total insertion size of ∼3.6 kb and a construct size of ∼4.4 kb (including homology arms) (Figure 3A). To optimize the generation of CKI c-JUN+B7-H3 CAR T cells, we utilized the genome editing buffer (Thermo) for electroporation. Foy et al. implemented a manufacturing process relying on a precommercial version of the CTS Xenon electroporator (Thermo), which has been described to enhance editing efficiencies and cell yields.^30^ We tested 4 independent donors and confirmed the feasibility of our process, this time utilizing the new 4.4-kb construct. We observed the expected decrease in T cell viability 1 day after the electroporation, followed by recovery (Figure 3B). We observed improved baseline CKI efficiencies (21%–31.6%) (Figures 3C and 3D) and total cell yields with the new electroporation buffer, resulting in an ∼3-fold expansion of target cells over electroporated cells by day 14 (Figure 3E). Interestingly, the 1-kb difference in insert size between B7-H3 CAR and Jun+ B7-H3 CAR did not affect the final CAR T cell counts.

To provide functional evidence that the co-insertion of c-JUN enhances CKI B7-H3 CAR T cell activity in antigen-low SCLC, we assessed their performance *in vitro* via co-culture against the antigen-low H524 cell line (2,172 molecules/cell) and found improved secretion of IL-2 and IFNγ via ELISA (Figures 3F, 3G, S3A, and S3B). Next, we tested c-JUN+B7-H3 CAR T cells *in vivo* against H524 xenograft models and observed a significant tumor growth reduction on day 16 post-tumor cell injection (*p* = 0.027) (Figures 3H and 3I).

### Clinical-scale manufacture of B7-H3 CKI CAR T cells is efficient and results in products with a favorable activity profile

**Figure 4:**
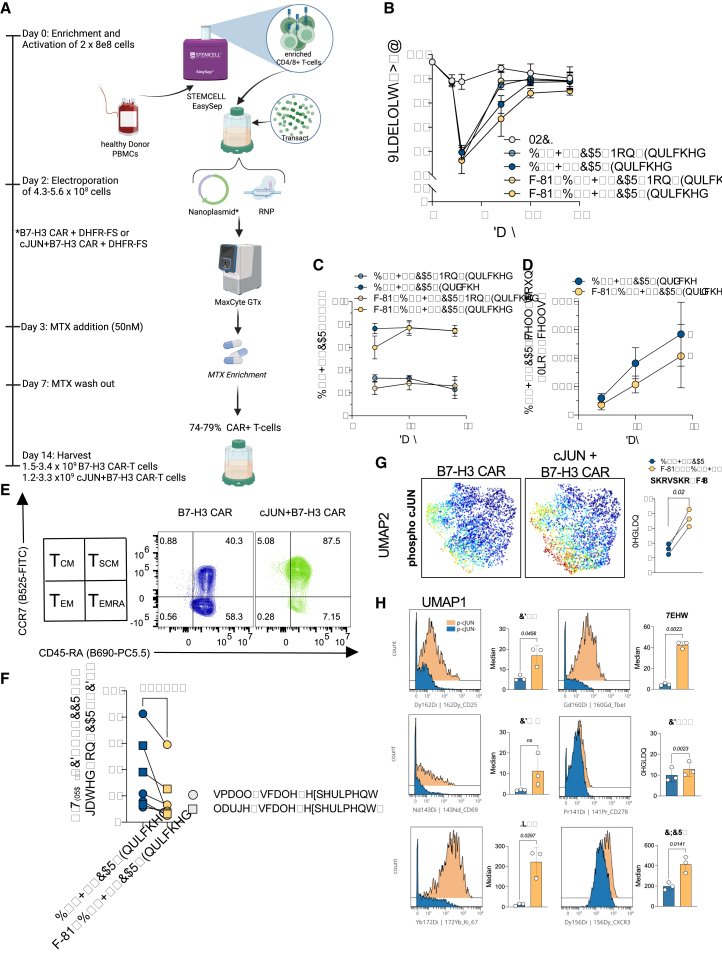
Clinical-scale manufacturing and in-depth characterization of CRISPR knockin c-JUN+B7-H3 CAR T cells (A) Schematic overview of process workflow. (B) In-process assessment of cell viability over time. (C) CAR knockin and enrichment efficiencies across three independent donors tested at clinical scale. (D) Total CAR T cell counts from both enriched large-scale cultures over time (*n* = 3 independent donors). Of note, a culture split was conducted on day 10. Therefore, day 14 counts are extrapolated (∗). (E) Representative quadrant plot from one donor for memory markers CCR7 and CD45RA (gated on CAR+ T cells). (F) Frequency of T_EMRA_ within CAR+ T cell populations in final products from large-scale cultures and small-scale experiments from Figure 3 (*n* = 7 independent donors). All samples were obtained on day 14. (G) Uniform manifold approximation and projection (UMAP) plot from mass cytometry analysis of large-scale products (*n* = 3 donors, left) and median expression levels of phospho-*c*-JUN across all three donors tested (right). (H) Histograms (left) and bar plots (right) comparing levels of CD25, CD69, Ki67, T-bet, CD278, and CXCR3 in phospho-c-JUN-positive versus -negative cells from c-JUN+B7-H3 CAR T cells (*n* = 3 donors). Statistical differences were assessed using two-tailed and paired *t* tests. Error bars indicate the SD.

Given our encouraging results of successfully generating functional c-JUN+B7-H3 CAR T cells, we next addressed the challenge of scaling up our pipeline. To this end, we processed Leukopaks from three independent healthy donors and scaled up the electroporation reaction in a linear manner, aiming for a factor of 25×. We utilized the Maxcyte GTx electroporator and the GMP-compatible CL1.1 single-shot cartridge (Figure 4A). In line with our small-scale experiments, we observed a relevant decline in total T cell viability 1 day post-electroporation. However, non-enriched and enriched samples recovered by days 7 and 10 post-activation, respectively (Figure 4B). Again, we observed average insertion efficiencies of 29.6% with the B7-H3 CAR and 26.2% with the c-JUN+B7-H3 CAR construct, respectively. Enrichment post-CEMENT across all three donors and both B7-H3 CAR constructs resulted in a 3- to 3.5-fold CAR enrichment (Figure 4C). Furthermore, on average, our approach resulted in a 3.6-fold expansion of CAR+ T cells over electroporated T cells for CKI B7-H3 CAR T cells and a 2.8-fold expansion of c-JUN+B7-H3 CAR T cells over electroporated T cells by day 14 (Figure S4A). We obtained cell counts from in-process samples of enriched large-scale cultures to calculate target cell yields, which confirmed CAR T cell growth over time. Moreover, in all donors and across both constructs, we successfully manufactured 1.2–3.5 × 10^9^ CAR+ T cells by day 14, which is sufficient to provide multiple CAR T cell doses (Figure 4D).

Phenotypic analysis of in-process samples and final products via flow cytometry revealed that CD4/8 ratios declined over time in CAR+ T cells and without relevant differences across all samples (Figure S4B). In addition, only donor-to-donor differences were observed for exhaustion markers when tested on final products (Figure S4C). However, we identified a significantly reduced frequency of CCR7-/CD45RA + T_EMRA_ cells in large- and small-scale samples of enriched c-JUN+B7-H3 CAR T cells as compared to enriched B7-H3 CAR T cells (average +c-JUN = 17.8% versus average −c-JUN = 34.5%) (Figures 4E and 4F). Of note, we observed a significant increase in central memory (T_CM_) and effector memory (T_EM_) populations when comparing c-JUN-positive with c-JUN-negative CKI B7-H3 CAR T cells and no consistent changes in the memory stem cell (T_SCM_) subset (Figure S4D). To gain in-depth insights into the functional state of CKI c-JUN+B7-H3 CAR T cells compared to CKI B7-H3 CAR T cells, we performed mass cytometry of samples derived from our large-scale experiments. As expected, CKI c-JUN+B7-H3 CAR T cells expressed higher levels of phosphorylated c-JUN and a trend toward enhanced proliferative profiles, as determined by higher CD25 and Ki67 (Figures 4G and S4E). In contrast, we found downregulation of CD38, which plays an inhibitory role in activated T cells via regulation of glycolysis^47^ (Figure S4E). We further examined our mass-cytometry-derived high-dimensional dataset to assess the functional differences in CKI c-JUN+B7-H3 CAR T cells with high levels of phospho-c-JUN versus cells with low levels of phospho-c-JUN. In this context, we observed not only higher levels of markers of activation and proliferation (CD25, Ki67, and CD69) but also enhanced T-bet expression, which drives long-term renewal of memory CD8^+^ T cells toward effector function, increased expression of the co-stimulatory molecule CD278, and higher levels of CXCR3, which has been described to modulate effector differentiation^48^ (Figure 4H).

Our data thus far demonstrate that the generation of CKI c-JUN+B7-H3 CAR T cells at the clinical scale is feasible, and their phenotypic profiling indicates small but significant differences compared to CKI B7-H3 CAR T cells, in favor of a proliferative central memory and stem cell memory phenotype.

### CKI c-JUN+B7-H3 CAR T cells show no evidence of genotoxicity

**Figure 5:**
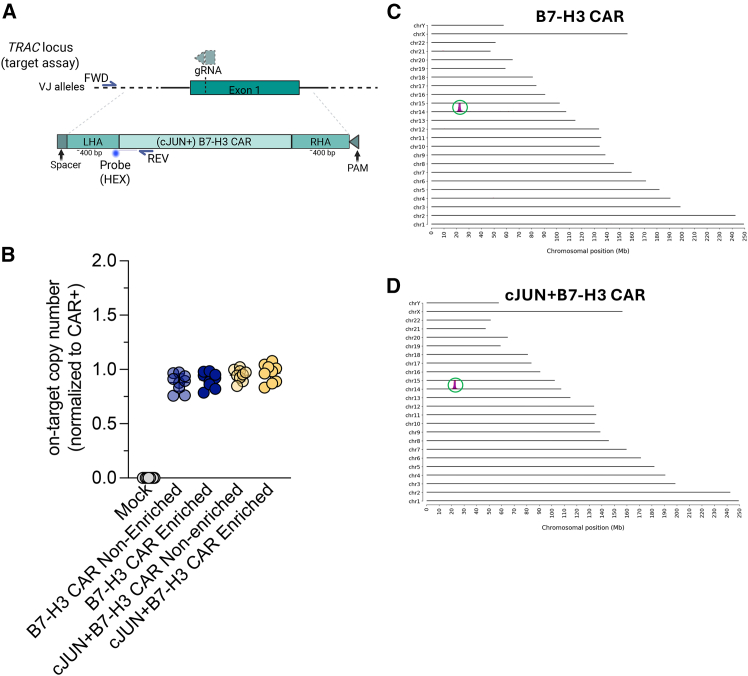
Genomic characterization of clinical-scale products (A) Design of a ddPCR assay to determine CAR copy numbers at the on-target site (*TRAC*). (B) Copy number of final products from Figure 4 obtained via ddPCR. Shown here are technical triplicates from *n* = 3 independent donors. (C and D) Insertion site analysis via targeted locus amplification for anti-B7-H3 CAR T cells (C) and c-JUN+B7-H3 CAR T cells (D) inserted into *TRAC* on chromosome 14. Results were obtained from the first donor of the large-scale runs shown in Figure 4.

To characterize the final products obtained from the large-scale manufacturing pipeline at the genomic level, we first established a droplet digital PCR (ddPCR) assay for the on-target copy-number (CN) quantification of CKI B7-H3 CAR constructs inserted into the *TRAC* locus. Primers were designed to bind within *TRAC* but outside the homology arm (forward) and within the poly(A) signal (reverse) (Figure 5A). After normalizing obtained raw CNs to the albumin reference standard (CN = 2) and the frequency of CAR+ T cells via flow cytometry, we identified CKI of B7-H3 CARs to result in an approximate CN of 1. This result indicates mono-allelic knockin across all tested samples (Figures 5B and S5). Next, we confirmed that our approach indeed resulted in targeted insertion of B7-H3 CAR and c-JUN+B7-H3 CAR. To this end, we performed targeted locus amplification. Here, we found both constructs to yield a single peak in *TRAC*, which is located on chromosome 14 (Figures 5C and 5D).

### TGF-β1 induces resistance against B7-H3 CAR T cells in thoracic SMARCA4-deficient UT

**Figure 6:**
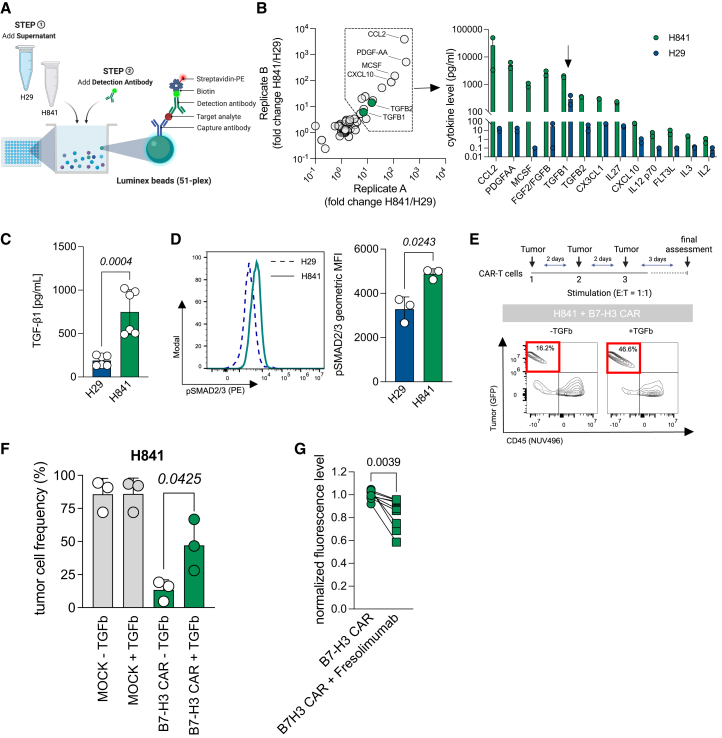
Identification of TGF-β1 as a key driver of resistance against B7-H3 CAR T cells in thoracic SMARCA4-deficient UTs (A) Experimental layout of a Luminex-based multiplex assay to screen a total of 51 cytokines in supernatant samples from NJH29 and H841 cell lines obtained from biological duplicates after 24 h of serum starvation. (B) Dot plot showing fold changes of cytokine secretion in H841 over NJH29 cells from two replicate experiments. Highlighted are the top hits (left). Absolute cytokine levels from top hit cytokines (right). (C) TGF-β1 secretion in NJH29 and H841 after 24 h of serum starvation assessed via ELISA (from *n* = 2 independent experiments, performed in triplicate). Statistical differences were assessed using two-tailed and paired *t* tests. (D) Expression of phospho-SMAD2/3 in NJH29 and H841 cell lines after 24 h of serum starvation. (Left) Representative histogram. (Right) Pooled data from three independent experiments. Statistical differences were assessed using an unpaired *t* test with Welch’s correction. (E) Repeated stimulation of non-viral B7-H3 CAR T cells with H841 cells in either the presence or the absence of TGF-β1 (10 ng/mL). (Top) Experimental overview. (Bottom) Representative quadrant plot indicating tumor cell frequency in the upper left quadrant. (F) Pooled data (from *n* = 3 independent T cell donors). Statistical differences were assessed using two-tailed and paired *t* tests. (G) Normalized tumor cell viability of GFP+ H841 after co-culture at a 1:1 ratio in either the absence or the presence of the TGF-β neutralizing antibody fresolimumab (*n* = 3 independent donors). Error bars indicate the SD.

Differences in the susceptibility to B7-H3 CAR T killing by H841 and NJH29 cell lines presenting similar densities of B7-H3 on their surface (Figures 1, 2, and S2E–S2G) led us to hypothesize that thoracic SMARCA4-deficient UTs may secrete inhibitory molecules that interfere with CAR T activity. To test this hypothesis, we analyzed the secretome of NJH29 (B7-H3 CAR-sensitive) and H841 (B7-H3 CAR-resistant) cells using a bead-based multiplex cytokine assay covering 51 relevant human cytokines (Figure 6A). Among the top hits of differentially secreted cytokines in H841 versus NJH29 cells was the T cell-suppressive cytokine TGF-β1^49^ (Figure 6B). We further confirmed enhanced TGF-β1 expression in H841 versus NJH29 cells via ELISA (Figure 6C) and significantly higher baseline levels of SMAD2/3 phosphorylation in H841 cells (Figure 6D). To investigate the T cell-suppressive function of TGF-β1 in our system, we performed repeated rounds of co-culture of CKI B7-H3 CAR T cells against H841 cells in either the absence or the presence of TGF-β1. After three rounds of stimulation, co-cultures were assayed via flow cytometry. Indeed, we identified 4-fold higher tumor cell frequencies when adding TGF-β1 to the co-culture (Figures 6E and 6F). On the other hand, the addition of the TGF-β neutralizing antibody fresolimumab^50^ resulted in enhanced tumor cell killing when CKI B7-H3 CAR T cells were co-cultured with H841 cells (Figures 6G and S6A).

### c-JUN co-expression induces a combined type 1/2 profile and boosts efficacy of CKI anti-B7-H3 CAR T cells in thoracic SMARCA4-deficient UT

**Figure 7:**
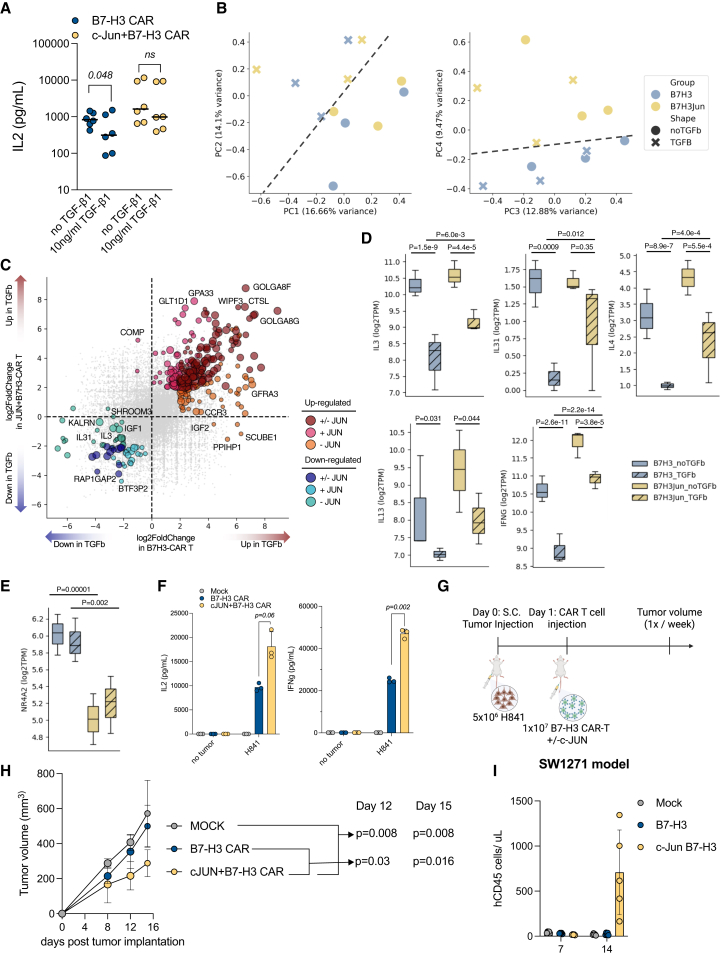
CKI c-JUN+B7-H3 CAR T cells overcome TGF-β1-mediated suppression in thoracic SMARCA4-deficient UTs (A) IL-2 secretion after stimulation with plate-bound B7-H3 antigen in the presence or absence of 10 ng/mL TGF-β1 (*n* = 3 donors, technical duplicates). (B–E) Transcriptomic analysis from samples described in (A). (B) Principal-component analysis (PCA) of indicated samples. (C) Scatterplot comparing differentially expressed genes in CKI anti-B7H3 CAR T cells ± c-Jun co-expression after TGF-β1 treatment. (D and E) Boxplots of selected cytokine genes (D) and *NR4A2* (E). The *p* values were generated by DESeq2. (F) IL-2 (left) and IFNγ (right) secretion after 24 h of co-culture at an E:T ratio of 1:1 in H841 cells. One out of six representative donors is shown. Statistical differences were assessed using two-tailed and paired *t* tests. Pooled data for all six donors are provided in Figures S7A and S7B. (G and H) Experimental setup for *in vivo* treatment of H841-derived xenograft tumors (G) and tumor volume over time post-tumor implantation (H). (I) Number of total human CD45^+^ T cells from peripheral blood obtained on days 7 and 14 after the first round of (CAR) T cell treatment of the SW1271 xenograft model. Related to Figures S7C–S7G. Statistical differences were assessed using a two-tailed Mann-Whitney *U*-test. Error bars indicate the SD.

The enhanced performance of CKI c-JUN+B7-H3 CAR T cells against antigen-low SCLC, as well as their favorable memory and activity profile, encouraged us to determine whether CKI c-JUN+B7-H3 CAR T cells can overcome TGF-β1-induced suppression. To this end, we stimulated CKI CAR T cells with plate-bound antigen in the presence or absence of TGF-β1. Here, we observed significantly reduced IL-2 secretion upon the addition of TGF-β1 in CKI B7-H3 CAR T cells (*p* = 0.048) and non-significant differences in CKI c-JUN+B7-H3 CAR T cells (Figure 7A). Encouraged by these findings, we performed bulk transcriptomic analysis via RNA sequencing (RNA-seq). First, we performed a principal-component analysis (PCA), and despite donor-to-donor variability, we observed that samples were distributed along PC1 and PC2 based on TGF-β1 treatment, while c-Jun co-expression resulted in sample separation alongside PC4 (Figure 7B). Next, we analyzed differential gene expression and observed that in CKI c-JUN+B7-H3 CAR T cells, the addition of TGF-β1 resulted in less downregulation of *IL3* and *IL31* (Figure 7C). When we expanded our analysis to additional type 2 cytokines *IL4* and *IL13*, as well as the type 1 cytokine *IFNG*, we observed the same trend (Figure 7D). Together, the expression of type 2 cytokines *IL3*, *IL31*, *IL4*, and *IL13* in anti-CD19 CAR T cells has recently been shown to associate with improved long-term outcome in patients with B-ALL. Importantly, the same study highlighted that CAR T cell products with enhanced IFNγ secretion and tumor-killing capacity can be generated from patients with a poor response when their T cells are primed in media containing IL-4.^51^ Finally, we also observed strong downregulation of the transcription factor NR4A2 in c-Jun co-expressing T cells, which is known to regulate T cell homoeostasis, and its loss has been associated with enhanced anti-tumor activity and increased persistence in murine T cells (Figure 7E).^52^^,^^53^

Next, we tested CAR T cell products from large-scale manufacturing against H841 cells. As previously observed with small-scale products in low-antigen SCLC, we found increased secretion of both IL-2 and IFNγ in CKI B7-H3 CAR T cells co-expressing c-JUN (Figures 7F, S7A, and S7B). *In vivo* treatment of H841 xenografts demonstrated a strong benefit resulting from c-JUN co-insertion, with an average tumor reduction of 40% (*p* = 0.01) by day 15 post-tumor cell injection as compared to CKI B7-H3 CAR T cells without c-JUN co-expression (Figures 7G and 7H). Finally, in an independent xenograft model of thoracic SMARCA4-deficient UTs utilizing the SW1271 cell line, we observed similar tumor control by B7-H3 CAR T cells with or without c-Jun in a rechallenge model (Figures S7C and S7D), but further analysis of blood and spleens confirmed enhanced expansion, non-significant benefits in persistence, and diminished formation of T_EMRA_ cells in CKI c-JUN+B7-H3 CAR T cells (Figures 7I and S7E–S7G).

## Discussion

Here, we identify *CD276* overexpression in human SCLC as well as thoracic SMARCA4-deficient UTs and successfully develop a non-viral approach for targeted insertion of a B7-H3 CAR via CKI. In SCLC, our data highlight the importance of B7-H3 as an immunotherapeutic target, which is expressed at heterogeneous levels, that is still amenable to CAR-induced killing. A recent study analyzing B7-H3, B7-H4, and PD-L1 expression in 90 primary human SCLC samples identified the expression of B7-H3 in 64.9% of cases,^15^ supporting our finding obtained from transcriptomic data in an independent cohort,^2^ where *CD276* is robustly expressed independent of the NE and non-NE subtypes and at higher levels than other relevant targets (*DLL3* and *SEZ6*). Importantly, in transcriptomic data from longitudinally analyzed murine MYC-driven autochthonous SCLC (RPM model),^41^ *Cd276* expression again is not restricted to NE SCLC, which is a limitation of previously investigated CAR targets in SCLC.^8^^,^^9^

However, when CKI B7-H3 CAR T cells are challenged by lowering the E:T ratio within co-culture assays, we observe that the thoracic SMARCA4-deficient UT cell line H841 is resistant to killing and limits cytokine secretion. Following up on this result in a cytokine screen, we find that TGF-β1 is a differentially secreted hit in H841 cells when compared to NJH29 SCLC cells. TGF-β1 is known for its role in inducing T cell exhaustion^49^^,^^54^; however, its capability to limit therapeutic T cell function in thoracic SMARCA4-deficient UTs has not been described yet. In a repeated stimulation assay, we show that TGF-β1 dramatically improved tumor cell viability in the thoracic SMARCA4-deficient UT cell line H841, indicating the relevance of TGF-β1-induced exhaustion in our model of thoracic SMARCA4-deficient UTs.

We have recently shown that c-JUN overexpression renders CAR T cells resistant against exhaustion, a dysfunctional cell state commonly observed in T cells targeting solid tumors,^46^ and that c-JUN overexpression can overcome antigen heterogeneity.^35^ Thus, in the present study, we engineered B7-H3 CAR T cells to co-express the AP-1 factor c-JUN to increase T cell function in both SCLC with low antigen density and thoracic SMARCA4-deficient UTs expressing the T cell-suppressive cytokine TGF-β1. In the antigen-low SCLC cell line H524 (2,172 molecules/cell), we provide evidence both *in vitro* and *in vivo* that, in line with our previous findings, c-JUN co-expression can surpass B7-H3 antigen heterogeneity in SCLC.

To overcome TGF-β1-mediated signaling, mainly two cell engineering approaches have been explored for CAR T cells so far: disruption of *TGFBR2* via CRISPR knockout^49^ and expression of a dominant-negative TGF-β receptor 2.^55^ We demonstrate that our non-viral CKI approach to insert the anti-B7-H3 CAR along with c-JUN is not only translatable at the clinical scale but also results in products with enhanced activity, including expression of type 2 cytokines, which have been identified in anti-CD19 CAR T cells from patients with B-ALL with long-term responses of at least 5–8 years. Here, CAR T cells with type 2 cytokine expression were identified to alleviate dysfunction in other CAR T cell populations,^51^ thus providing an elegant explanation for how c-JUN co-expression enhances CAR T cell function in our system despite the presence of TGF-β1. Moreover, c-JUN co-expression results in improved CAR T cell expansion, persistence with less exhaustion, and improved tumor control *in vivo* in both SCLC with low antigen levels and thoracic SMARCA4-deficient UTs. This is an exciting finding, since previous studies utilized viral vectors to achieve c-Jun overexpression, resulting in enhanced CAR T cell function.^46^^,^^56^ However, with viral transduction, the gene dosage cannot be fully controlled. Here, we show that only one copy of c-Jun delivered to the *TRAC* locus is sufficient to drive small but significant transcriptional changes that result in enhanced persistence and potency. Finally, clinical-scale products manufactured in this study do not show any evidence of genotoxicity.

Manufacturing of CAR T cells relies on *ex vivo* cultivation and transduction with retro- or lentiviral vector for (semi-)random transgene integration.^57^ In contrast, CKI provides an exciting avenue for the manufacture of CAR T cells due to targeted genomic insertion. Here, the transgene is commonly delivered via adeno-associated virus type 6 (AAV6).^25^^,^^58^ However, the manufacture of any clinical-grade viral vector is cumbersome and expensive,^25^ preventing broad application and resulting in limited accessibility of CAR T cells for patients with SCLC and thoracic SMARCA4-deficient UTs. To overcome limited access to clinical vectors, we and others have previously proposed the utilization of naked and NP DNA templates for CKI, which can be manufactured at GMP grade.^26^^,^^27^^,^^28^^,^^30^ Importantly, non-viral CAR T cells generated via CKI have been shown to be non-inferior when compared to viral transduced CAR T cells previously.^28^^,^^59^

In the present study, we successfully inserted an ∼3.6-kb-sized construct and efficiently generated (c-JUN+)B7-H3 CAR T cells at the clinical scale. Of note, the full template, including homology arms, has a size of ∼4.4 kb, i.e., almost reaching the payload size limits for both AAV6 and ssDNA.^25^^,^^27^ Although non-viral insertion utilizing DNA templates is associated with potentially dose-limiting toxicity,^26^ our approach is robust, yielding 1.2–3.5 × 10^9^ CAR T cells within a 14-day process, which can be potentially shortened to a 10-day process.

In summary, our work provides a promising therapeutic opportunity for patients with SCLC and thoracic SMARCA4-deficient UTs that can be swiftly translated for clinical testing. In addition, B7-H3 is being investigated as a CAR T cell target in various tumor entities,^14^ indicating the broad scope of our non-viral genome engineering approach, even beyond the treatment of SCLC and thoracic SMARCA4-deficient UTs.

### Limitations of the study

Throughout this study, we utilized SCLC and SMARCA4-deficient UT cell lines, which do not faithfully recapitulate roadblocks of immunotherapy arising from the complex tumor microenvironment in solid tumors. Furthermore, the utilization of xenograft models in immunodeficient mice limited the study of long-term functionality of c-JUN co-expressing B7-H3 CAR T cells due to the graft versus host effect. Addressing these points in an immunocompetent mouse model would require re-engineering of a mouse-specific B7-H3 CAR, since the scFv currently in clinical trials does not cross-react. Also, the genetic disease background of murine SCLC models is limited in comparison to cell line libraries. Ultimately, clinical trials are needed to test B7-H3 CAR T cells with c-JUN co-expression in both SCLC and SMARCA4-deficient UTs.

## Resource availability

### Lead contact

Further information and requests for resources and reagents should be directed to and will be fulfilled by the lead contact, Steven A. Feldman (feldmans@stanford.edu).

### Materials availability

All NP DNA templates generated during this study are available upon reasonable request and a materials transfer agreement.

### Data and code availability

• Bulk RNA-seq data generated in this study have been deposited in the Gene Expression Omnibus (GEO) repository and are available under the identifier GEO: GSE307259.
• This paper does not report original code.
• Any additional information required to reanalyze the data reported in this paper is available from the lead contact upon reasonable request.

## Acknowledgments

H.B.-W. is supported by the 10.13039/501100001659German Research Foundation (#555464052), the Ministerium für Kultur und Wissenschaft des Landes Nordrhein-Westfalen within the Cancer Research Center Cologne Essen (CCCE), and the 10.13039/501100009983Center for Molecular Medicine Cologne (CAP36). D.S. was supported by an MD Research Stipend of the Else Kröner Forschungskolleg Clonal Evolution in Cancer, University Hospital Cologne, Cologne, Germany. This work was supported by grants from the 10.13039/100000002NIH (R35CA283888, C.L.M.), the Mark Foundation Endeavor Award (J.S., C.L.M., and E.S.), the US NIH (C.L.M. and S.A.F.), the St. Baldrick’s Foundation EPICC Team (Empowering Pediatric Immunotherapies for Childhood Cancers; C.L.M.), the 10.13039/100014547Parker Institute for Cancer Immunotherapy (C.L.M.), and the 10.13039/100006352Virginia and D.K. Ludwig Fund for Cancer Research (C.L.M.).

## Author contributions

H.B.-W., E.S., C.L.M., and S.A.F. conceived and designed this study. H.B.-W., V.K., M.D.C.A., M.M., D.D.K., P.X., K.H., K.A., J.H., A.R., S.P., C.F., N.G.-L., Q.J., X.L., and S.H. executed the experiments and analyzed the data. Y.C., D.S., L.M., R.U., J.G., R.T., J.S., E.S., C.L.M., and S.A.F. analyzed the data. H.B.-W., E.S., V.K., C.L.M., and S.A.F. wrote the manuscript, with all authors contributing to writing and providing feedback.

## Declaration of interests

H.B.-W. received research funding from Wilson Wolf Manufacturing, LLC. C.L.M. is a founder of, equity holder in, and consultant for Link Cell Therapies and has received royalties from Juno and CARGO through NIH for CD22-CAR; consulting fees from CARGO, Ensoma, Astra Zeneca, Immatics, RedTree Venture Capital, Grace Science, Kite Pharma, and Nektar; and research funding from Tune Therapeutics. E.S. consults for Galaria LLC, Lepton Pharmaceuticals, and Cell.co. S.A.F. serves on the scientific advisory boards for Achieve Clinics, MFX, Autolomous, and Advanced Cell Therapy Centre (Oslo University Hospital); serves as a board member for Biotech Partners and Act for Hope; and has patents in the field of cell therapy and receives royalties from NIH related to those patents.

## STAR★Methods

### Key resources table

**Table:** 

REAGENT or RESOURCE	SOURCE	IDENTIFIER
**Antibodies**		

Zombie Aqua™ Fixable Viability Kit	Biolegend	cat. no. #423102; RRID: NA
Brilliant Violet 421™ anti-human TCR α/β Antibody	Biolegend	cat. no. #306722; RRID:AB_2562805
Recombinant Human B7-H3 Fc Chimera Protein, CF	R&D systems	cat. no. #1027-B3-100; RRID: NA
Human B7-H3 PE-conjugated Antibody	R&D systems	cat. no. #FAB1027P; RRID:AB_2073697
BD OptiBuild™ BUV737 Mouse Anti-Human CD4	BD Biosciences	cat. no. #750977; RRID:AB_2713927
BD Horizon™ BUV805 Mouse Anti-Human CD8	BD Biosciences	cat. no. #564912; RRID:AB_2833078
BD OptiBuild™ BUV496 Mouse Anti-Human CD45	BD Biosciences	cat. no. #750179; RRID:AB_2868405
PerCP anti-human CD45RA Antibody	Biolegend	cat. no. #304156; RRID:AB_2616997
FITC anti-human CD197 (CCR7) Antibody	Biolegend	cat. no. #353216; RRID:AB_10916386
FITC anti-human CD39 Antibody	Biolegend	cat. no. #328206; RRID:AB_940425
BD Pharmingen™ PE-Cy™7 Mouse anti-Human CD279 (PD-1)	BD Biosciences	cat. no. 561272; RRID:AB_10611585
Brilliant Violet 421™ anti-human CD223 (LAG-3) Antibody	Biolegend	cat. no. #369314; RRID:AB_2629797
PE anti-human CD366 (Tim-3) Antibody	Biolegend	cat. no. #345006; RRID:AB_2116576
PE Mouse anti-Smad2 (pS465/pS467)/Smad3 (pS423/pS425)	BD Biosciences	cat. no. #562586; RRID:AB_11151915
phospho c-JUN (S73)	Abcam	cat. no. #ab250081; RRID: NA
Purified Mouse Anti-Human CD25	BD Biosciences	cat. no. #555430; RRID:AB_395824
Anti-Human/Mouse T-bet (4B10)-160Gd	Standard BioTools	cat no.# 3160010B; RRID:AB_2810251
Purified anti-human CD69 (Maxpar(R) Ready)	BioLegend	cat no.# 310939; RRID:AB_2562827
Purified Mouse Anti-Human CD278	BD Biosciences	cat. no. #557801; RRID:AB_396877
Anti-Cross Ki-67 (B56)-172Yb	Standard BioTools	cat. no. #3172024B; RRID:AB_2858243
Anti-Human CD183/CXCR3 (G025H7)-156Gd	Standard BioTools	cat. no. #3156004B; RRID:AB_2687646
Purified anti-human CD38 (Maxpar® Ready) Antibody	BioLegend	cat. no. #303535; RRID:AB_2562819

**Biological samples**		

Leukapheresis of healthy donors	Stemcell Technologies	N/A

**Chemicals, peptides, and recombinant proteins**		

BD QuantiBRITE PE Beads	BD Biosciences	Cat# 340495
Dynabeads Human T-Expander CD3/CD28	Thermo Fischer Scientific, Gibco	Cat# 11141D
Human IL-7	Miltenyi Biotec	Cat# 130-095-367
Human IL-15	Miltenyi Biotec	Cat# 130-095-760
TexMACS™ Medium	Miltenyi Biotec	Cat# 130-097-196
T cell TransAct™, human	Miltenyi Biotec	Cat# 130-111-160
CliniMACS CD8 GMP MicroBeads	Miltenyi Biotec	Cat# 170-076-703
CliniMACS CD4 GMP MicroBeads	Miltenyi Biotec	Cat# 170-076-702
Lipofectamine 2000 Transfection Reagent	Thermo Fischer Scientific	Cat# 11668500
RetroNectin Recombinant HumanFibronectin Fragment	Takara	Cat# T100B
Human Male AB Plasma-Derived Serum, Heat Inactivated (Human Thrombin)	Access Biologicals	Cat# 535-Hl
CryoStor CS10	STEMCELL Technologies	Cat# 07930
Alt-R.Sp. Cas9 protein	IDT	Cat# 1072532
CTS™ Xenon™ Genome Editing Buffer	Thermo Fisher Scientific	Cat# A4998001
Electroporation Buffer, Small	MaxCyte	Cat# EPB-1
Nexcelom Bioscience Llc AO/PI Viability stain 5mL	Fisher Scientific	Cat# NC0285242
Corning™ Matrigel™ Matrix	Fisher Scientific	Cat# CB-40234
eBioscience™ 1× RBC Lysepuffer	BD Biosciences	Cat# 00-4333-57
Cisplatin	Fluidigm	Cat# 201064
Q™ Four Element Calibration Beads	Fluidigm	Cat #201078
Cell-ID™ Intercalator-Ir	Standard BioTools	Cat. #201192B
MAXPAR Cell Staining Buffer (CSB)	Standard BioTools	Cat. #201068
MAXPAR Fix/Perm Buffer	Standard BioTools	Cat. #NC0987611
Fresolilumab	Selleckchem	Cat. #A2336
Biotinylated Human B7-H3/CD276 Protein, Fc,Avitag™ (MALS verified)	Acro Biosystems	Cat. #B73-H82F5
Recombinant Human TGF-beta 1 Protein	R&D Systems™	Cat. #18339417

**Critical commercial assays**		

Human IFN-g ELISA MAX Deluxe	BioLegend	Cat #430104
Human IL-2 ELISA MAX Deluxe	BioLegend	Cat #431804
Intracellular Fixation & Permeabilization Buffer Set Kit	BD Biosciences	Cat #88-8824-00
Dylight 650 Micoscale Antibody Labeling Kit	Thermo Fischer Scientific	Cat #84536
RosetteSep Human T cell Enrichment Cocktail	STEMCELL Technologies	Cat #15061
SepMate-50 Tubes	STEMCELL Technologies	Cat #85450
REAlease® CD4/CD8 (TIL) MicroBead Kit, human	Miltenyi	Cat #130-121-561
RNeasy Micro kit	Qiagen	Cat #74104
Maxpar® X8 Multimetal Labeling Kit	Standard BioTools	Cat #201300
LEGEND MAX™ Free Active TGF-β1 ELISA Kit	BioLegend	Cat #437707
PureLink Genomic DNA Mini Kit	Invitrogen	Cat #K182001
ddPCR Supermix for probes (no dUTP)	BioRad	Cat. #1863024

**Deposited data**		

Human SCLC processed RNA-Seq data	George et al.^2^	https://doi.org/10.1038/nature14664.
Human thoracic SMARCA4 deficient UT RNA-Seq data	Le Loarer et al.^40^	https://doi.org/10.1038/ng.3399.
Murine SCLC RNA-Seq from longitudinal samples of the RPM model	Ireland et al.^41^	https://doi.org/10.1016/j.ccell.2020.05.001.
CKI B7-H3 CAR-T cells +/− c-JUN treated with TGF-b1	this manuscript	Gene Expression Omnibus (GEO): GSE307259

**Experimental models: Cell lines**		

CORL-88	Dr. Roman Thomas	RRID:CVCL_1141
H1836	Sos et al.^37^	RRID:CVCL_1498
DMS-273	Dr. Roman Thomas	RRID:CVCL_1176
H1930	Dr. Roman Thomas	RRID:CVCL_1507
GLC-1	Sos et al.^37^	RRID:CVCL_8200
H2171	Sos et al.^37^	RRID:CVCL_1536
GLC-8	Sos et al.^37^	RRID:CVCL_8218
N417	Sos et al.^37^	RRID:CVCL_1602
H29	Jahchan et al.^45^	established in Jahchan et al.
SBC-4	Sos et al.^37^	RRID:CVCL_A479
H69	Dr. Roman Thomas	RRID:CVCL_1579
SBC-6	Sos et al.^37^	RRID:CVCL_DH97
H82	Sos et al.^37^	RRID:CVCL_1591
SCLC-22H	Dr. Roman Thomas	RRID:CVCL_2186
H146	Sos et al.^37^	RRID:CVCL_1473
SW1271	Sos et al.^37^	RRID:CVCL_1716
H187	Dr. Roman Thomas	RRID:CVCL_1501
H211	Sos et al.^37^	RRID:CVCL_1529
H524	Sos et al.^37^	RRID:CVCL_1568
H526	Sos et al.^37^	RRID:CVCL_1569
H841	Sos et al.^37^	RRID:CVCL_1595
H889	Sos et al.^37^	RRID:CVCL_1598
H1092	Sos et al.^37^	RRID:CVCL_1454

**Experimental models: Organisms/strains**		

Mice: NSG (NOD.Cg-Prkdcscid Il2rgtm1Wjl/SzJ)	The Jackson Laboratory	Cat# JAX:005557; RRID:IMSR_JAX:005557

**Oligonucleotides**		

TRAC-CAR Probe (5′-3′): TAACGCGGCCGCTCCCCAGCATGCC (HEX)	this manuscript	N/A
TRAC-CAR FWD (5′-3′): CTGGGGTTTTGAAGAAGATCC	this manuscript	N/A
TRAC-CAR REV (5′-3′): GGGAGGATTGGGAAGAGA	this manuscript	N/A
Albumin Probe (5′-3′): CCTGTCATGCCCACACAAATCTCTCC (FAM)	this manuscript	N/A
Albumin FWD (5′-3′): CTGTCATCTCTTGTGGGCTGT	this manuscript	N/A
Albumin REV (5′-3′): ACTCATGGGAGCTGCTGGTT	this manuscript	N/A
sgRNA targeting TRAC: 5′-GGGAATCAAAATCGGTGAATAGG -3	Kath et al.^60^	https://doi.org/10.1016/j.omtm.2022.03.018

**Recombinant DNA**		

B7-H3_CAR_HITI	this manuscript	N/A
B7-H3_CAR_HDR	this manuscript	N/A
B7-H3_CAR+DHFR_HDR	this manuscript	N/A
cJUN+B7-H3_CAR+DHFR_HDR	this manuscript	N/A
CAR.CD19.FMC63.8aHTM.BBz	Majzner et al.^43^	N/A
CAR.B7H3.MGA271.28HTM.BBz	Majzner et al.^43^	N/A
RD114	Heitzeneder et al.^35^	N/A

**Software and algorithms**		

Excel	Microsoft Corp.	N/A
GraphPad Prism v8.4	GraphPad Software Inc.	N/A
FlowJo v10.7.1	FlowJo, LLC	N/A
Living Image version (IVIS imaging)	Perkin Elmer	N/A
SnapGene v5.2.3	SnapGene	N/A
OMIQ	OMIQ	N/A
Biorender	Biorender	N/A
SPICE	Roederer et al.^61^	https://doi.org/10.1002/cyto.a.21015
STAR aligner	Dobin et al.^62^	https://doi.org/10.1093/bioinformatics/bts635
Gencode v36	Frankish et al.^63^	https://doi.org/10.1093/nar/gkac1071
DESeq2	Love et al.^64^	https://doi.org/10.1186/s13059-014-0550-8

**Other**		

Incucyte ZOOM	Sartorius	N/A
Beckman CytoFLEX analyzer	Beckman Coulter	N/A
ExPERT GTx™ GMP Electroporator	MaxCyte	N/A
OC-100 × 2	MaxCyte	N/A
CL1.1 RUO	MaxCyte	N/A
Cellometer Auto 2000	Nexcelom	N/A
G-Rex®6 Well Plate	Wilson Wolf Manufacturing	Cat# 80240M
G-Rex®100M Open System, Research Use Only	Wilson Wolf Manufacturing	Cat# RU81100
BD LSRFortessa™ X-20	BD Biosciences	N/A
gentleMACS dissociator	Miltenyi	N/A
Helios mass cytometer	Fluidigm	N/A
Streptavidin Coated - 96 well Solid plates - Clear PS	Biozol	Cat. #BMT-MC0STF-SA5/200
QX200 AutoDG	BioRad	N/A
QX200 Droplet Reader	BioRad	N/A

### Experimental model and study participant details

#### Isolation of human CD4/CD8 enriched T-cells from leukopaks

Human Leukopaks derived from healthy donors were ordered from STEMCELL Technologies. To enrich for CD4/8 positive T-cells we used the EasySep Human T cell Isolation Kit (STEMCELL Technologies). Enriched T-cells were aliquoted and frozen for small-scale experiments or directly activated for large-scale experiments (see below). No statistical method was used to predetermine samples size. Sample sizes are similar to those reported in previous publications.^28^^,^^35^^,^^46^ Sex was not considered as a biological variable in our analysis.

#### Tumor cell lines

Cell lines used in this study have been recently described and were obtained from ATCC or established in our laboratories or elsewhere.^13^^,^^37^^,^^38^ Cell lines were cultured either in RPMI or HITES media, which was supplemented with 10–20% FBS, 1% Penicillin/Streptomycin and 1% L-glutamine, identified through STR profiling and tested for mycoplasma contamination.

#### *In vivo* animal studies

NOD-SCID-γc^−/−^ (NSG) mice were purchased from the Jackson Laboratory and all animal studies were carried out according to Stanford University Animal Care and Use Committee–approved protocols in a barrier facility (APLAC protocol #31287). Mice were bred in house (Stanford University) in accordance to APLAC guidelines, as described previously.^35^ Healthy male and female mice were used for *in vivo* experiments between 6 and 10 weeks old at tumor engraftment. Animals were housed in sterile cages in a barrier facility at Stanford University with a 12-h light/dark cycle and were monitored by the Veterinary Services Center (VSC) at Stanford University. Prior to subcutaneous injection into the flank of NSG mice, tumor cell lines NJH29, H524, SW1271 and H841 were resuspended in PBS. Treatment with indicated numbers of CAR-T cells was conducted via intravenous injection into the tail vein. Tumor volume was measured via caliper measurement using the formula: Width^2^ x Length x 0.5 = tumor volume. Peripheral blood was collected via retro-orbital puncture under isoflurane anesthesia at the indicated time point. Samples (50 μL) were processed by labeling for CD45, CD4, and CD8, lysed using BD FACS Lysing Solution (10× Concentrate, BD Biosciences) and analyzed on an LSR Fortessa X-20. Mice were euthanized when they presented symptoms or exceeded tumor growth as designated in the APLAC-approved protocols. Spleens in indicated experiments were harvested and mechanically dissociated via the gentleMACS dissociator (Miltenyi). After dissociation samples were passed through a 70μm cell strainer, depleted for red blood cells by ACK lysis, and filtrated by applying flow cytometry filter tubes with 35μm cell strainer caps (Falcon) yielding in a single-cell suspension. The REAlease CD4/CD8 (TIL) MicroBead Kit (Miltenyi) was applied according to the manufacturer’s instructions to enrich for T cells.

### Method details

#### Cell counts and viability

All samples were counted using the Nexcelom Cellometer Auto 2000. To ensure accurate discrimination between living and dead cells, we stained samples with AO/PI dye at a 1:1 volume ratio. Hereafter, samples were analyzed using the setting: Immune Cells – Low RBC.

#### Viral transduction of CAR-T cells

To generate viral transduced B7-H3 CAR-T cells, we activated frozen enriched human T-cells using Dynabeads for activation via CD3/CD28 (Thermo Fisher) at a 1:1 ratio and cultivated cells in TexMACS media supplemented with human IL-7 and IL-15 each at 12.5ng/mL (Miltenyi Biotec) and containing 3% human AB Serum (Access Cell Culture). Viral CAR constructs and protocols for transduction have been previously established and for the B7-H3 CARs were designed as follows: MGA271 (anti-B7-H3 scFv) -CD28TM - 4-1BB - CD3z.^43^ Briefly, retroviral vectors were spinoculated on Retronectin (Takara) and human T-cells were added on days 1 and 2 post activation. Dynabeads were removed the following day and T-cells were cultivated until day 9.

#### Design and synthesis of CKI templates

Synthetic genes were custom synthesized at Genscript and shipped to Aldevron. For downstream cloning into nanoplasmid DNA (Aldevron), construct ends were flanked by NheI and KpnI cut sites respectively. The B7-H3 CAR utilized for CKI experiments was designed analogous to the viral B7-H3 CAR. After manufacture, nanoplasmid DNA was formulated in H_2_O at a concentration of 3mg/mL.

#### Small-scale and large-scale CRISPR knock-in

Human CD4/CD8 enriched T-cells were activated using research grade Transact (Miltenyi Biotec) and cultivated in G-Rex (Wilson Wolf). On day 2 post activation, cells were harvested and electroporated utilizing the Maxcyte GTx. Per electroporation at small scale, 2.5 μL of wildtype Cas9 were complexed with a *TRAC* targeting sgRNA^60^: 5′-GGGAATCAAAATCGGTGAAT**AGG** -3’ (both IDT) for 10 min. Hereafter, 0.75 μg of nanoplasmid DNA per 1 × 10^6^ T-cells were added to the RNP for 10 min at room temperature. 1–2x10^7^ T-cells were either resuspended in 100 μL of Maxcyte Buffer (Maxcyte/Cytiva) or Genome Editing Buffer (Thermo Fisher) and added to the RNP/DNA mix. Samples were electroporated using the OC-100 × 2 assembly (Maxcyte). For large-scale electroporation, samples were again harvested on day 2 post activation and resuspended in Genome Editing Buffer at a final concentration of 2 × 10^8^/mL. The volume of RNP and nanoplasmid DNA was adjusted to the volume of electroporation buffer. Per milliliter of electroporation buffer we prepared 50 μL of RNP and 50 μL nanoplasmid DNA. Hereafter, samples were electroporated using the GMP compatible CL1.1 assembly. All electroporations were conducted using the program Expanded TC-4.

#### CRISPR knock-in EnrichMENT (CEMENT)

To efficiently enrich for CAR+ T-cells post editing, we applied CEMENT. CEMENT relies on the co-expression of a mutant version of the Dihydrofolate Reductase enzyme, which confers resistance against the clinically approved drug Methotrexate. We recently optimized the start (day 3 post activation) and duration (4 days) of the enrichment schedule^28^ and applied our optimized platform to the study presented here.

#### Flow cytometry and quantibrite

Samples were stained for flow cytometry as described previously.^28^ To assess antigen density levels via Quantibrite (BD Biosciences), an additional sample containing Quantibrite beads was analyzed according to manufacturer instructions as described previously.^36^ Intracellular staining of phospho-SMAD2/3 was performed on serum starved (24 h) samples, which were fixed for 50 min at 4°C and permeabilized at 4°C using the Fixation/Permeabilization Solution Kit (BD Biosciences). All samples were assessed on the CytoFLEX LX (Beckman Coulter).

#### Mass cytometry

For mass cytometry, 1 × 10^6^ frozen cells were washed with PBS twice and resuspended in PBS containing cisplatin (250nM, Fluidigm) to assess viable cells. After incubation for 3 min at room temperature, cells were washed with cell staining medium (CSM, 1× PBS with 0.05% BSA, 0.02% sodium azide), fixed with 1.6% paraformaldehyde diluted in PBS for 10 min at RT, and then washed with PBS. Samples were subsequently frozen. Upon thawing and washing in CSM, barcoding was performed, and samples were pooled. A master mix of titrated surface antibodies was prepared, filtered (0.1 mm) and then added to the pooled and barcoded sample for 30 min at RT. Following surface staining, samples were washed twice in CSM and permeabilized with ice-cold methanol for 10 min on ice, then washed again twice in CSM. Samples were stained with titered intracellular antibodies for 45 min on ice followed by 2 subsequent washes with CSM. Finally, samples were resuspended in DNA intercalator (Fluidigm, 1:5000 191Ir/193Ir and 1% PFA in 1× PBS) and incubated overnight at 4°C for next day acquisition (Helios). On the day of acquisition, samples were washed once in CSM and twice in filtered ddH_2_O. Cells were then resuspended at 1×10^6^ cells/mL in ddH2O with 1× EQ four-element beads (Fluidigm Corporation, no. 201078). Cells were acquired on a Fluidigm Helios mass cytometer.

#### *In vitro* killing and ELISA assays

CAR-T cells were harvested on indicated days of culture and co-cultured in 96-well plates at a 1:1, 1:5 and 1:10 (effector to target) cell ratio based on the number of CAR+ T-cells and using 50,000 tumor cells per well. Total volumes in all co-culture assays performed in 96-well plates were kept at 200 μL. For live cell imaging-based assessment of GFP+ tumor cell viability over time we employed the Incucyte device (Sartorius). To block TGF-β1 signaling the TGF-β neutralizing antibody Fresolimumab (Selleckchem) was used at a final concentration of 50nM. To obtain supernatants for IL-2 and IFNγ ELISA assays (BioLegend), we co-cultured 50,000 tumor cells with 50,000 CAR+ T-cells and collected supernatants 24 h later. In case of plate-bound stimulation biotinylated B7-H3 antigen (Acro Biosystems) was dissolved in PBS resulting in a final concentration of 500ng/mL, and 100 μL per well were added onto streptavidin coated 96-well plates (Eagle Biosciences) over night. Next day, antigen was removed, and plates were washed. Hereafter, 50.000 CAR^+^ T cells were added per well in the presence or absence of TGF-β1 as indicated. For the TGF-β1 ELISA (BioLegend) we serum starved tumor cells for 24 h. Samples were processed according to manufacturer’s instructions and plates were analyzed using the Varioskan Lux (Thermo Scientific).

#### Repeated stimulation

We plated 1 × 10^6^ tumor cells and added either 1 × 10^6^ MOCK or B7-H3 CAR-T cells. TGF-β1 (R&D Systems) was added at a final concentration of 10ng/mL. On indicated time points T-cells were harvested, counted and added to freshly plated tumor cells at a 1:1 ratio either in the presence or absence of TGF-β1. On the day of final assessment samples were analyzed for GFP expression (tumor cells) and CD45 expression (T cells).

#### Luminex

Cell lines were plated in serum free media overnight and supernatant was harvested after 24 h of incubation. Samples were stored at −80 and transferred to the Human Immune Monitoring Center (HIMC) at Stanford. The multiplex cytokine assay was conducted utilizing the human 48-plex (EMD Millipore) and TGF-β1 (Merck) panels. A detailed protocol can be found here: https://iti.stanford.edu/himc/protocols.html. For downstream analysis, background values were averaged, and mean values were subtracted from values obtained in experimental samples. Samples with values being lower than the background control were set to 0. Next, fold changes of cytokine secretion in H841 over NJH29 cells were calculated.

#### ddPCR and copy number quantification

Final products from large scale experiments were used to extract genomic DNA (PureLink Genomic DNA Mini Kit, Invitrogen). Next, samples were prepared according to the instructions of the Bio-Rad ddPCR Supermix for probes (no dUTP) protocol. We used 40ng of genomic DNA and performed an in-ddPCR digestion via HindIII-HF. To assess the on-target CAR copy-number, we established a ddPCR assay utilizing a primer pair, which consist of a forward primer to bind within the *TRAC* locus, but outside the homology arm sequence, and a reverse primer to bind within the poly-A sequence of our knock-in templates. A previously described assay to determine the Albumin copies per sample and the CAR% identified via flow cytometry were used to calculate the normalized CAR copy number as described previously.^28^ The QX200 AutoDG (Bio-Rad) was employed to generate droplets and samples were analyzed post PCR using the QX200 Droplet Reader (Bio-Rad). The QX software enabled assessment of samples and manual threshold was used to clearly separate positive and negative droplets. The following primers and probes (IDT) were used in this study for ddPCR:

TRAC-CAR Probe (5′-3′): TAACGCGGCCGCTCCCCAGCATGCC (HEX);

TRAC-CAR FWD (5′-3′): CTGGGGTTTTGAAGAAGATCC;

TRAC-CAR REV (5′-3′): GGGAGGATTGGGAAGAGA;

Albumin Probe (5′-3′): CCTGTCATGCCCACACAAATCTCTCC (FAM);

Albumin FWD (5′-3′): CTGTCATCTCTTGTGGGCTGT;

Albumin REV (5′-3′): ACTCATGGGAGCTGCTGGTT.

#### Insertion site analysis

Targeted locus amplification enabled the unbiased identification of B7-H3 CAR and c-JUN+B7-H3 CAR insertion sites across the whole genome. To this end, 5–10 × 10^6^ T-cells derived from final products of the first large-scale donor were frozen and shipped to Cergentis B.V. for targeted locus amplification, which includes digestions, reverse crosslinking with ligation, PCR and subsequent sequencing. NGS reads were aligned to the CAR sequence and the human genome (hg19 sequence) as described previously.^28^

#### RNA extraction, sequencing and processing

CAR T cells were derived from the plate-bound stimulation assay described above and collected after ∼20 h of incubation. Cells were counted to ensure a maximum number of 5 × 10^5^ per sample. Next, cell pellets were obtained after centrifugation and processed using the Qiagen RNeasy Micro kit and following the manufacturer’s protocol. Bulk RNAseq was performed by Novogene using the NovaSeq platform. The raw RNA sequencing data was mapped to human reference genome hg38 using the STAR aligner,^62^ and genes annotated in Gencode v36^63^ was quantified using featurecounts in the subread package.^65^ The differential gene expression analysis was conducted in the DESeq2 package,^64^ and significant differentially expressed genes were filtered by absolute log2FoldChange above 2 and adjusted *p*-value below 0.05. Data visualization was generated using python.

#### Analysis of published transcriptomic data

Transcriptomic data derived from primary human SCLC^2^ and thoracic SMARCA4-deficient UT^40^ have been published previously, and FPKM or TPM values respectively were log_2_ transformed for normalization and subsequent comparison of immunomodulatory ligand expression. For analysis of transcriptomic data derived from the RPM mouse model, which was previously described by Ireland et al., log_2_ normalized data was downloaded from NCBI GEO: GSE149180.

### Quantification and statistical analysis

#### Statistical analysis and software

Data was analyzed using Microsoft Excel and GraphPad Prism. The number of independent T cell donors, technical replicates and statistical tests utilized to analyze data are described in the respective figure legends. Flow cytometry derived.fcs files were analyzed with FlowJo (FlowJo LLC). Sequences for sgRNAs and nanoplasmid DNA templates were visualized with SnapGene (Dotmatics). SPICE analysis to assess expression of exhaustion markers in final products was performed as described elsewhere.^61^ Mass cytometry data was analyzed using OMIQ software. For schematic illustrations we used BioRender.

## Supplemental information

Document S1. Figures S1–S7

Document S2. Article plus supplemental information
