# Supplementary material for: c-JUN enhances CRISPR knockin anti-B7-H3 CAR T cell function in small cell lung cancer and thoracic SMARCA4-deficient undifferentiated tumors
Source: Cell Rep Med. 2026 Jan 20;7(1):102549. doi: 10.1016/j.xcrm.2025.102549 (PMC12866126; doi:10.1016/j.xcrm.2025.102549)
Supplement: Document S1. Figures S1–S7 [file mmc1.pdf]

Cell Reports Medicine, Volume *7*

Supplemental information

c-JUN enhances CRISPR knockin anti-B7-H3 CAR T cell function in small cell lung cancer and thoracic SMARCA4-deficient undifferentiated tumors

Hyatt Balke-Want, Vimal Keerthi, Maria Del Carmen Arenas, Yiyun Chen, Meena Malipatlolla, Dorota D. Klysz, Peng Xu, Katie Ho, Kyle Asano, David Stahl, Jing Huang, Aidan Retherford, Sunny Patel, Carley Fowler, Lukas Maas, Nikolaos Gkitsas-Long, Qiaoshi Jiang, Xikun Liu, Roland Ullrich, Julie George, Sabine Heitzeneder, Ramya Tunuguntla, Julien Sage, Elena Sotillo, Crystal L. Mackall, and Steven A. Feldman

Figure S1

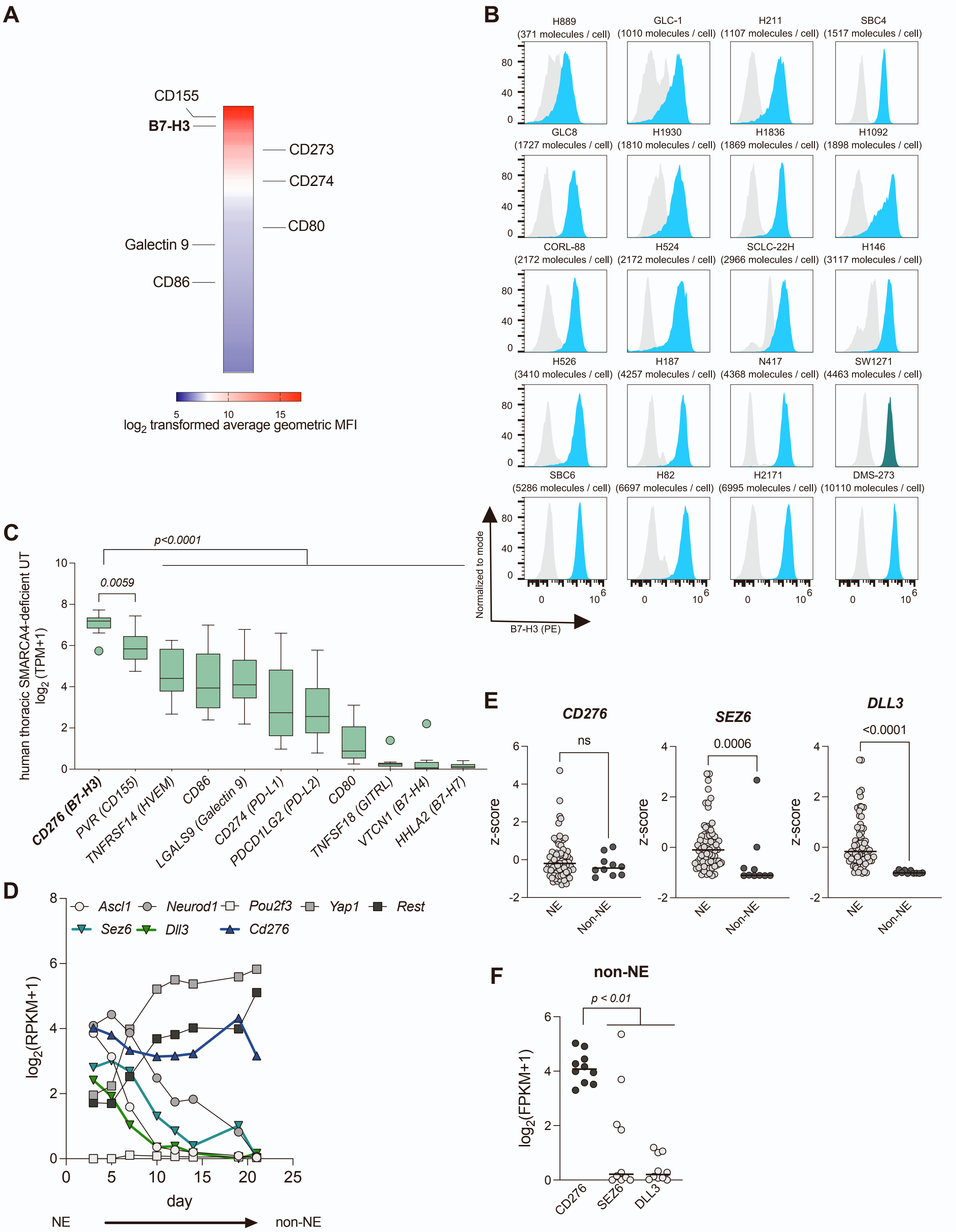
A
CD155
B7-H3
CD273
CD274
CD80
Galectin 9
CD86
5
10
15
log2 transformed average geometric MFI
B
H889 (371 molecules / cell)
GLC-1 (1010 molecules / cell)
H211 (1107 molecules / cell)
SBC4 (1517 molecules / cell)
GLC8 (1727 molecules / cell)
H1930 (1810 molecules / cell)
H1836 (1869 molecules / cell)
H1092 (1898 molecules / cell)
CORL-88 (2172 molecules / cell)
H524 (2172 molecules / cell)
SCLC-22H (2966 molecules / cell)
H146 (3117 molecules / cell)
H526 (3410 molecules / cell)
H187 (4257 molecules / cell)
N417 (4368 molecules / cell)
SW1271 (4463 molecules / cell)
SBC6 (5286 molecules / cell)
H82 (6697 molecules / cell)
H2171 (6995 molecules / cell)
DMS-273 (10110 molecules / cell)
Normalized to mode
B7-H3 (PE)
C
p<0.0001
0.0059
human thoracic SMARCA4-deficient UT
log2 (TPM+1)
CD276 (B7-H3)
PVR (CD155)
TNFRSF14 (HVEM)
CD86
LGALS9 (Galectin 9)
CD274 (PD-L1)
PDCD1LG2 (PD-L2)
CD80
TNFSF18 (GITRL)
VTCN1 (B7-H4)
HHLA2 (B7-H7)
D
Ascl1
Neurod1
Pou2f3
Yap1
Rest
Sez6
Dll3
Cd276
log2(RPKM+1)
day
NE
non-NE
E
CD276
SEZ6
DLL3
ns
0.0006
<0.0001
z-score
NE
Non-NE
F
non-NE
p < 0.01
log2(FPKM+1)
CD276
SEZ6
DLL3

Supplementary Figure 1: B7-H3 is expressed in human SCLC and thoracic SMARCA4-deficient UT at relevant levels

A, Pooled protein expression levels identified from screening 332 surface markers across n = 5 human SCLC cell lines from Weiskopf et al. Highlighted are relevant immunomodulatory ligands. B, B7-H3 expression and antigen density levels in human SCLC and thoracic SMARCA4-deficient UT cell lines screened in this study. Related to Figure 1B. C, RNA expression levels of relevant immunomodulatory ligands in primary human thoracic SMARCA4-deficient UT (from Le Loarer et al., *n=13*). Statistical differences were determined using 2-way ANOVA and Dunnett's multiple comparison test. D, RNA expression of key SCLC transcription factors as well as *Cd276*, *Dll3* and *Sez6* over time in the RPM mouse model. Original data has been previously published by Ireland et al. E, RNA expression levels (z-score) of indicated genes in NE (*ASCL1*$^{+}$ and/or *NEUROD1*$^{+}$) and non-NE (*POU2F3*$^{+}$ and/or *REST*$^{+}$) SCLC. Differences were evaluated for statistical significance by Mann-Whitney-U test. F, RNA expression of indicated genes within non-NE samples compared via Wilcoxon test. Primary data in E and F was obtained from George et al.

Figure S2

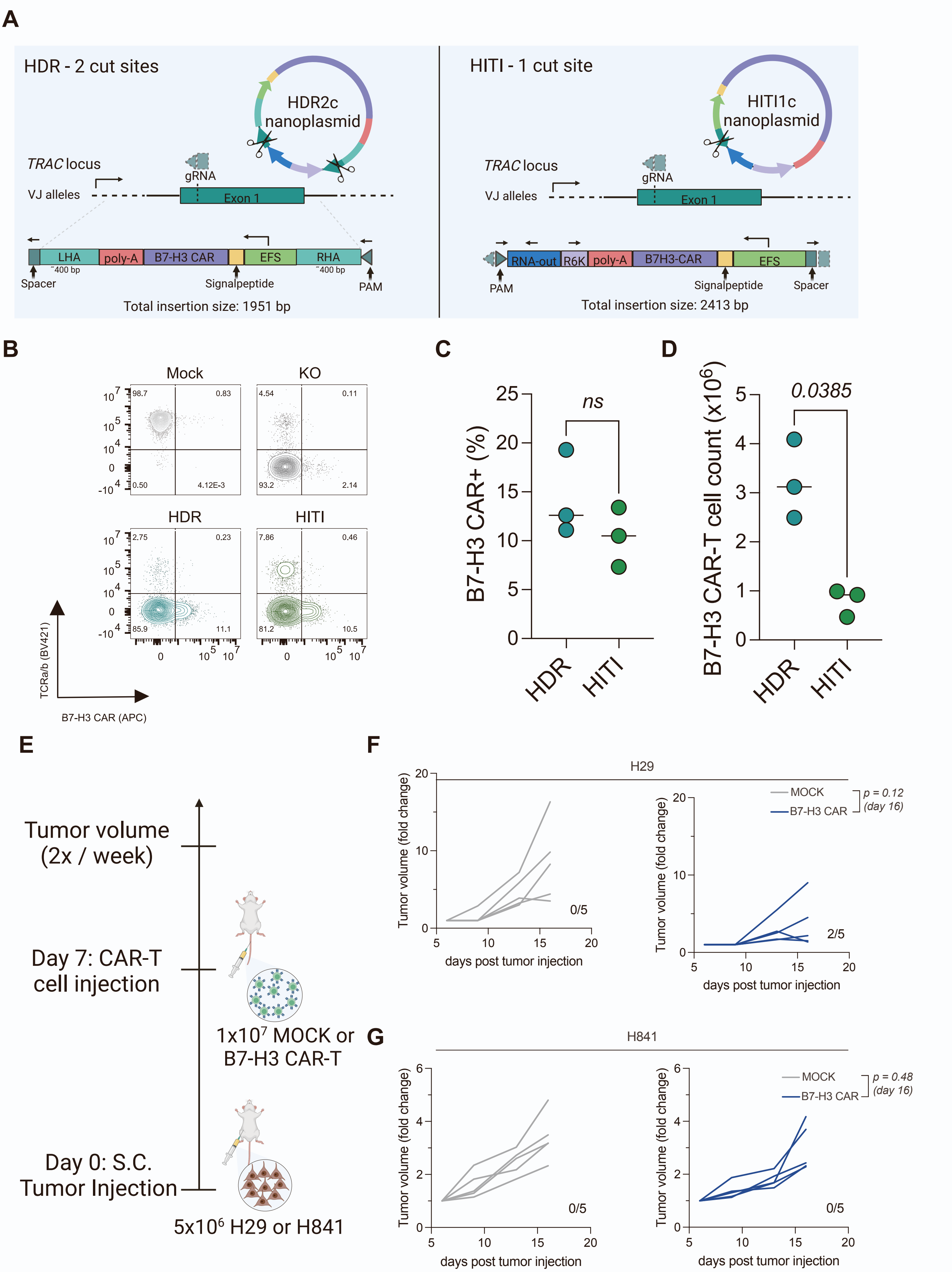
A
HDR - 2 cut sites
HDR2c nanoplasmid
TRAC locus
gRNA
VJ alleles
Exon 1
LHA
poly-A
B7-H3 CAR
EFS
RHA
~400 bp
~400 bp
Spacer
Signalpeptide
PAM
Total insertion size: 1951 bp
HITI - 1 cut site
HITI1c nanoplasmid
TRAC locus
gRNA
VJ alleles
Exon 1
RNA-out
R6K
poly-A
B7H3-CAR
EFS
PAM
Signalpeptide
Spacer
Total insertion size: 2413 bp
B
Mock
KO
HDR
HITI
98.7
0.83
0.50
4.12E-3
4.54
0.11
93.2
2.14
2.75
0.23
85.9
11.1
7.86
0.46
81.2
10.5
TCRa/b (BV421)
B7-H3 CAR (APC)
C
ns
B7-H3 CAR+ (%)
HDR
HITI
D
0.0385
B7-H3 CAR-T cell count (x10^6)
HDR
HITI
E
Tumor volume (2x / week)
Day 7: CAR-T cell injection
1x10^7 MOCK or B7-H3 CAR-T
Day 0: S.C. Tumor Injection
5x10^6 H29 or H841
F
H29
Tumor volume (fold change)
days post tumor injection
0/5
2/5
MOCK
B7-H3 CAR
p = 0.12 (day 16)
G
H841
Tumor volume (fold change)
days post tumor injection
0/5
0/5
MOCK
B7-H3 CAR
p = 0.48 (day 16)

Supplementary Figure 2: Optimization and in vivo testing of non-viral CRISPR knock-in anti-B7-H3 CAR-T cells

A, Schematic representation of NP constructs used for CKI of a B7-H3 CAR into *TRAC* either depending on HDR or HITI. B, Representative quadrant plot indicating fractions of CRISPR knock-out and knock-in populations. C+D, $1x10^7$ cells were electroporated on day 2 post activation using a total of 7.5µg of DNA. Knock-in frequencies (C) and yields (D) for B7H3 CAR+ T-cells as determined on day 10 post activation are shown (n = 3 donors). Differences were evaluated for statistical significance by paired, two-tailed t tests. E, Schematic overview of the in vivo experiment. F+G, Fold change of tumor volume over baseline measurement prior to T-cell injection in NJH29 (F) and H841 (G) cell lines. Indicated are mice with stable disease as defined by less than one tumor doubling over the monitored period. Differences on day 16 were evaluated for statistical significance by unpaired, two-tailed t tests.

Figure S3

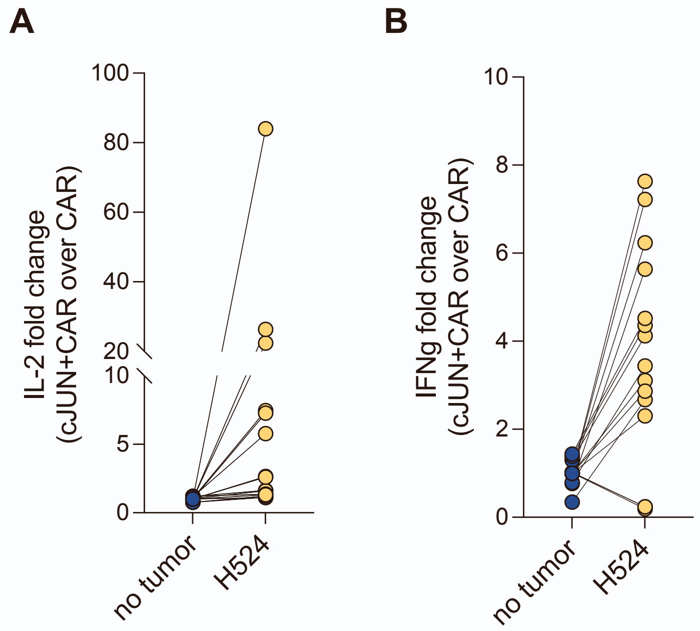

Supplementary Figure 3: Functional enhancement of non-viral CRISPR/Cas9 knock-in c-JUN+anti-B7H3 CAR T-cells in antigen low SCLC.

A+B, Pooled data across five independent donors for fold changes in IL-2 (A) and IFNγ (B) secretion of c-JUN+B7-H3 CAR-T cells over B7-H3 CAR-T cells without c-JUN co-expression. Related to Figure 3F+G.

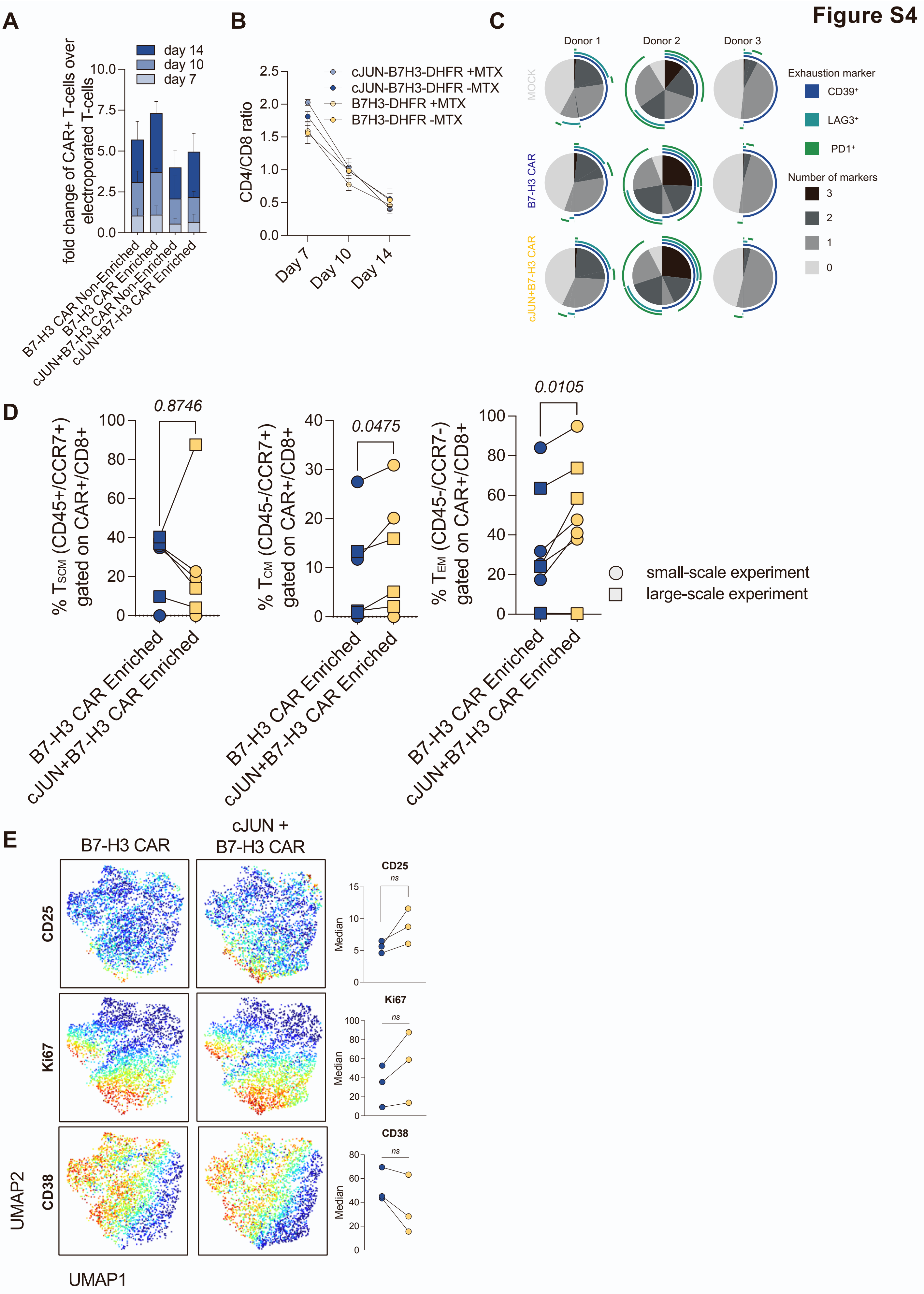
Figure S4
A
B
C
D
E
day 14
day 10
day 7
fold change of CAR+ T-cells over electroporated T-cells
B7-H3 CAR Non-Enriched
B7-H3 CAR Enriched
cJUN+B7-H3 CAR Non-Enriched
cJUN+B7-H3 CAR Enriched
cJUN-B7H3-DHFR +MTX
cJUN-B7H3-DHFR -MTX
B7H3-DHFR +MTX
B7H3-DHFR -MTX
CD4/CD8 ratio
Day 7
Day 10
Day 14
Donor 1
Donor 2
Donor 3
MOCK
B7-H3 CAR
cJUN+B7-H3 CAR
Exhaustion marker
CD39+
LAG3+
PD1+
Number of markers
3
2
1
0
0.8746
0.0475
0.0105
% TSCM (CD45+/CCR7+) gated on CAR+/CD8+
% TCM (CD45-/CCR7+) gated on CAR+/CD8+
% TEM (CD45-/CCR7-) gated on CAR+/CD8+
B7-H3 CAR Enriched
cJUN+B7-H3 CAR Enriched
small-scale experiment
large-scale experiment
B7-H3 CAR
cJUN + B7-H3 CAR
CD25
Ki67
CD38
ns
Median
UMAP2
UMAP1

Supplementary Figure 4: In-process and final product characterization

A, Fold change of total CAR-T cells counts on indicated process days over the number of electroporated T-cells on day 2 post activation (n = 3 independent donors). B, CD4/CD8 ratios of CAR+ T-cells at indicated time points. C, Exhaustion marker expression of CAR+ T-cells obtained from final products on day 14 (*n = 3* independent donors). D, Frequency of indicated memory subsets. *n = 7* independent donors. E, UMAP plots from mass cytometry analysis of large scale products (*n=3* donors, left) and median expression levels of indicated markers across all three donors tested (right).

Figure S5

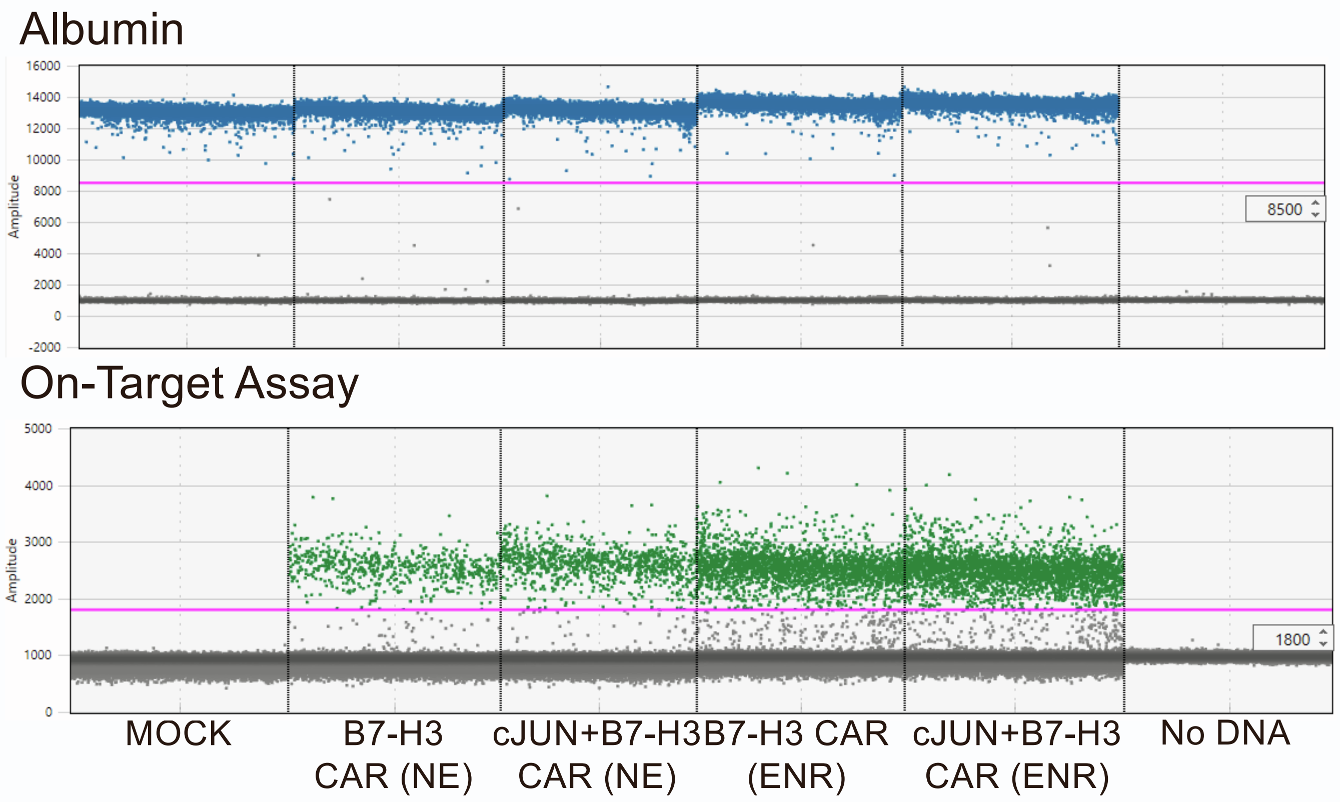

Supplementary Figure 5: Establishment of a ddPCR assay for precise copy number assessment at the on-target site

Amplitude levels for Albumin control assay and for B7-H3-CAR into *TRAC* test assay.

A

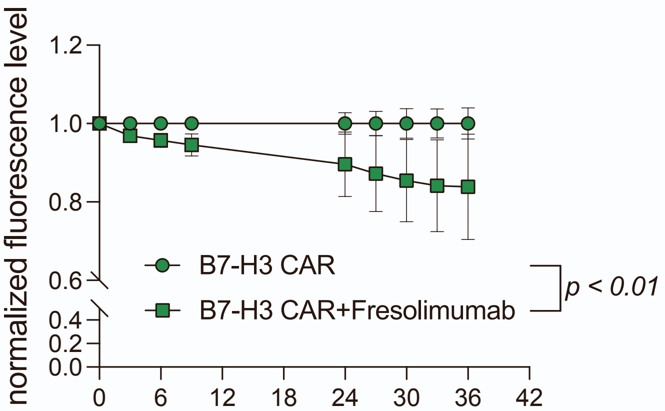

Supplementary Figure 6: Fresolimumab enhances CRISPR knock-in anti-B7-H3 CAR-T cell killing in thoracic SMARCA4-deficient UT

A, Time course of CKI anti-B7-H3 CAR-T cell killing in the presence or absence of Fresolimumab assessed via live cell imaging. Related to Figure 6G.

Figure S7

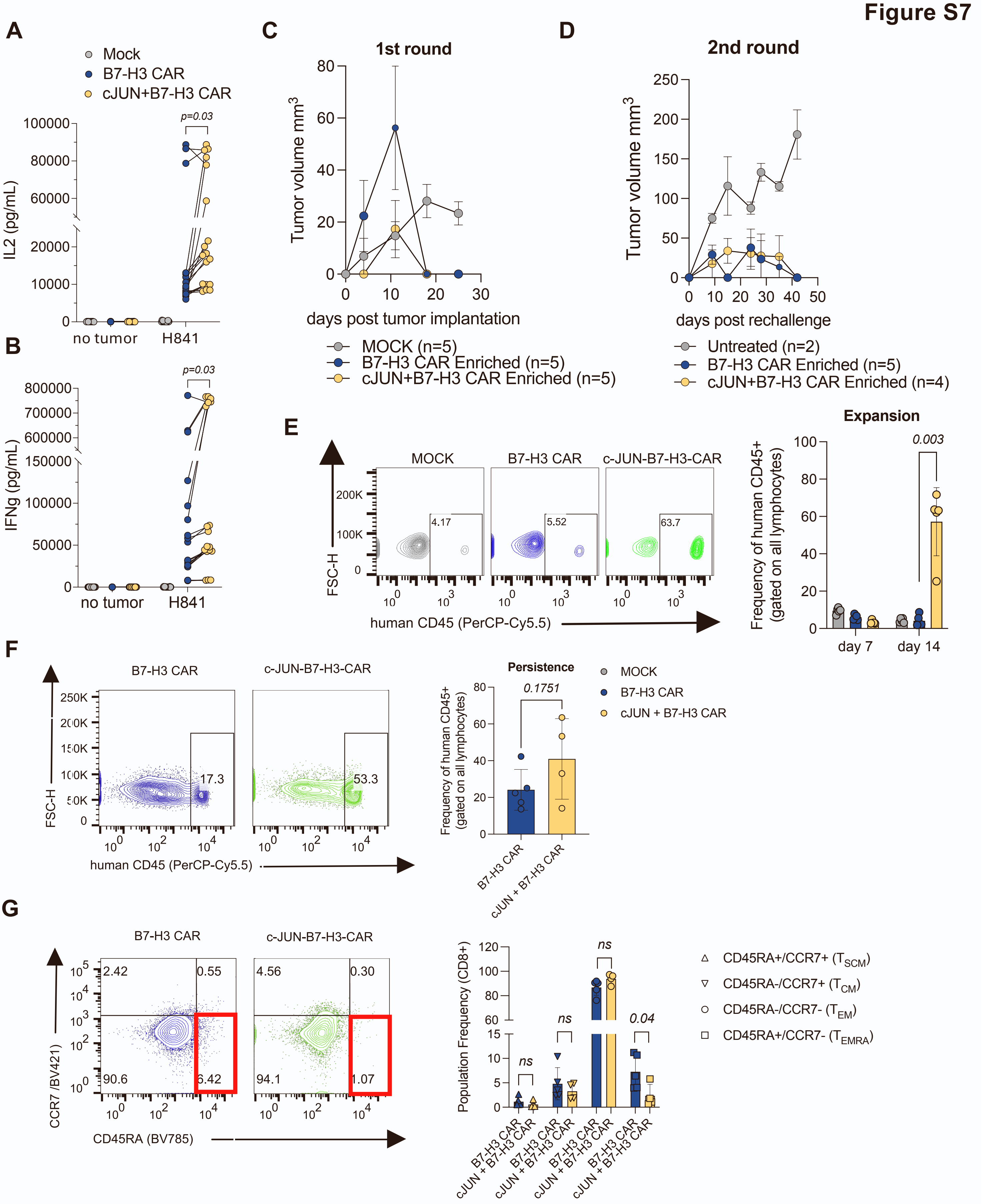
A
Mock
B7-H3 CAR
cJUN+B7-H3 CAR
p=0.03
IL2 (pg/mL)
100000
80000
60000
20000
10000
0
no tumor
H841
B
p=0.03
IFNg (pg/mL)
800000
700000
600000
150000
100000
50000
0
no tumor
H841
C
1st round
Tumor volume mm3
days post tumor implantation
MOCK (n=5)
B7-H3 CAR Enriched (n=5)
cJUN+B7-H3 CAR Enriched (n=5)
D
2nd round
Tumor volume mm3
days post rechallenge
Untreated (n=2)
B7-H3 CAR Enriched (n=5)
cJUN+B7-H3 CAR Enriched (n=4)
E
MOCK
B7-H3 CAR
c-JUN-B7-H3-CAR
200K
100K
4.17
5.52
63.7
FSC-H
human CD45 (PerCP-Cy5.5)
Expansion
0.003
Frequency of human CD45+
(gated on all lymphocytes)
day 7
day 14
F
B7-H3 CAR
c-JUN-B7-H3-CAR
250K
200K
150K
100K
50K
17.3
53.3
FSC-H
human CD45 (PerCP-Cy5.5)
Persistence
0.1751
Frequency of human CD45+
(gated on all lymphocytes)
B7-H3 CAR
cJUN + B7-H3 CAR
MOCK
B7-H3 CAR
cJUN + B7-H3 CAR
G
B7-H3 CAR
c-JUN-B7-H3-CAR
2.42
0.55
90.6
6.42
4.56
0.30
94.1
1.07
CCR7 /BV421)
CD45RA (BV785)
Population Frequency (CD8+)
ns
ns
ns
0.04
B7-H3 CAR
cJUN + B7-H3 CAR
CD45RA+/CCR7+ (TSCM)
CD45RA-/CCR7+ (TCM)
CD45RA-/CCR7- (TEM)
CD45RA+/CCR7- (TEMRA)

Supplementary Figure 7: CRISPR/Cas9 knock-in c-JUN+anti-B7H3 CAR T-cell activity in thoracic SMARCA4-deficient UT

A+B, Pooled data across six independent donors (3 at small and 3 at large scale) for IL-2 (A) and IFNγ (B) secretion at an E:T ratio of 1:1. Statistical differences were assessed using two-tailed and paired t-test. Error bars indicate SD. C, In vivo assessment of c-JUN+anti-B7H3 CAR T-cell in SW1271 xenograft models. $5x10^6$ SW1271 cells resuspended in PBS were injected into the right flank of each animal. On day 4 animals were treated with $1x10^7$ CAR+ T cells via tail vein injection. Mock treated animals were sacrifized at the end of the experiment due to graft versus host disease (GvHD). CKI CAR T cells do not express a T-cell receptor and therefore did not induce GvHD. D, CAR T cell treated animals were re-challenged with $1x10^6$ SW1271 cells resuspended in PBS:Matrigel (1:1). SW1271 cells were injected into the left flank of respective animals. Two additional animals received tumor cells to confirm successful tumor engraftment. One animal in the c-JUN-CAR arm was excluded from analysis due to unknown reason of death. Error bars indicate s.e.m. E, Representative quadrant plots showing relative frequencies of human CD45+ lymphocytes in peripheral blood obtained on day 14 after first round of treatment of SW1271 xenograft models with respective (CAR) T cells. Events were gated on lymphocytes and then on single cells (left). Summarized frequencies of human CD45+ lymphocytes on day 7 and day 14 after CAR T cell treatment (right). F+G, At the end of the experiment spleens were harvested to assess frequencies of human CD45+ lymphocytes (F) and their memory phenotype (G). Statistical differences were assessed using two-tailed and paired t-test. Error bars indicate SD.
